# Preparing to move: Setting initial conditions to simplify interactions with complex objects

**DOI:** 10.1371/journal.pcbi.1009597

**Published:** 2021-12-17

**Authors:** Rashida Nayeem, Salah Bazzi, Mohsen Sadeghi, Neville Hogan, Dagmar Sternad

**Affiliations:** 1 Department of Electrical and Computer Engineering, Northeastern University, Boston, Massachusetts, United States of America; 2 Department of Biology, Northeastern University, Boston, Massachusetts, United States of America; 3 Institute for Experiential Robotics, Northeastern University, Boston, Massachusetts, United States of America; 4 Departments of Mechanical Engineering and Brain and Cognitive Sciences, Massachusetts Institute of Technology, Cambridge, Massachusetts, United States of America; 5 Department of Physics, Northeastern University, Boston, Massachusetts, United States of America; Johns Hopkins University, UNITED STATES

## Abstract

Humans dexterously interact with a variety of objects, including those with complex internal dynamics. Even in the simple action of carrying a cup of coffee, the hand not only applies a force to the cup, but also indirectly to the liquid, which elicits complex reaction forces back on the hand. Due to underactuation and nonlinearity, the object’s dynamic response to an action sensitively depends on its initial state and can display unpredictable, even chaotic behavior. With the overarching hypothesis that subjects strive for predictable object-hand interactions, this study examined how subjects explored and prepared the dynamics of an object for subsequent execution of the target task. We specifically hypothesized that subjects find initial conditions that shorten the transients prior to reaching a stable and predictable steady state. Reaching a predictable steady state is desirable as it may reduce the need for online error corrections and facilitate feed forward control. Alternative hypotheses were that subjects seek to reduce effort, increase smoothness, and reduce risk of failure. Motivated by the task of ‘carrying a cup of coffee’, a simplified cup-and-ball model was implemented in a virtual environment. Human subjects interacted with this virtual object via a robotic manipulandum that provided force feedback. Subjects were encouraged to first explore and prepare the cup-and-ball before initiating a rhythmic movement at a specified frequency between two targets without losing the ball. Consistent with the hypotheses, subjects increased the predictability of interaction forces between hand and object and converged to a set of initial conditions followed by significantly decreased transients. The three alternative hypotheses were not supported. Surprisingly, the subjects’ strategy was more effortful and less smooth, unlike the observed behavior in simple reaching movements. Inverse dynamics of the cup-and-ball system and forward simulations with an impedance controller successfully described subjects’ behavior. The initial conditions chosen by the subjects in the experiment matched those that produced the most predictable interactions in simulation. These results present first support for the hypothesis that humans prepare the object to minimize transients and increase stability and, overall, the predictability of hand-object interactions.

## Author summary

Humans interact with objects that have complex dynamics every day. One example is picking up a cup filled with coffee to drink: the hand applies a force not only to the cup, but also indirectly to the liquid, which in turn acts back on the hand. These interactions can quickly become unpredictable, i.e., making it difficult for a person to control the potentially chaotic interactions in real-time. When starting such an action from rest, the object experiences start-up dynamics, or transients, that partly depend upon the initial conditions. We examined how subjects learn to identify initial conditions to shorten transients and make the ensuing interaction dynamics stable and predictable. Using a simplified model of the cup of coffee, a 2D cup with a rolling ball inside and moving on a horizontal line, subjects were encouraged to prepare the object dynamics to find the best initial conditions prior to starting a continuous transport task. Experimental and modeling results showed that with practice subjects converged to a small set of initial conditions, which decreased their transient durations, and achieved increasingly predictable interactions. The typical objectives in unconstrained tasks—to decrease effort and increase smoothness—could not be supported.

## Introduction

Functional interaction with objects—tool use—is essential in daily life and is regarded as a foundation of our evolutionary advantage. Manipulation of objects requires contact and interaction that present control challenges beyond those in unconstrained reaching and pointing that have been the mainstay of motor neuroscience. Studies of how humans manipulate objects have focused on tasks such as grasping, lifting, and transporting an object, often including analysis of the forces applied by the fingers onto the object [1–5]. However, typically in these studies the object is a rigid body, and the subject holds, transports, and places this rigid object from one point to another. This type of object manipulation is relatively simple compared to the variety of the interactions that occur in real life. Objects can have complex geometric configurations and internal degrees of freedom that give rise to complex interaction dynamics that are absent in simple transport and placing of solid objects. Examples of objects with complex interactions are painting with a paint brush, pouring water from a bottle, or leading a cup filled with coffee to one’s mouth to drink. In the latter example, the hand applies a force not only to the cup, but also indirectly to the liquid which, in turn, acts back onto the hand, potentially perturbing its trajectory. Therefore, the hand must make subtle adjustments to avoid spilling the coffee. The challenges of such complex interactions become apparent in individuals with neurological disorders, where small disturbances can easily lead to failure, making essential daily tasks, such as self-feeding or dressing, a difficult endeavor.

Relatively few studies in motor neuroscience have gone beyond rigid objects to explore the control strategies with more complex objects, i.e., interactions that involve intrinsic degrees of freedom that can create unstable object dynamics. The paradigms involved balancing a pole or transporting or compressing a mass spring system, where the controller must stabilize an inherently unstable system. In pole balancing, the classical control problem of stabilizing an inverted pendulum, human trajectories were modeled with a variety of controllers, such as intermittent, continuous, or predictive control with forward or inverse models [6–8]. Nonlinear time-series analysis of the human trajectory has focused on the role of noise and delays to distinguish between continuous or intermittent control [9–11]. One study examined the task of compressing a buckling spring and modeled human task performance with a simple dynamic model displaying a subcritical pitchfork bifurcation [12]. Another set of experiments examined how subjects transported a mass-spring system, an underactuated object with linear internal dynamics, to a target. These latter studies posited optimization criteria, such as generalized kinematic smoothness [13], minimum effort and maximum accuracy [14], and minimum acceleration with constraints on the center of mass [15]. However, while optimization approaches may be insightful for such highly controlled tasks, when interacting with more complex, nonlinear, and potentially chaotic object behavior, such optimization approaches will be challenged as prediction of this evolving dynamics is essential [16]. Hence, the question of how humans interact with objects with potentially unpredictable dynamics is still an open research problem.

To explore interaction with a dynamically complex object, our group has developed an experimental paradigm that investigated the transport of a ‘cup of coffee’, modeled as a ball rolling in a cup [17–19]. Implementing this model system in a virtual environment, subjects interact with the virtual object via a haptic robotic interface, moving the cup on a horizontal line; the rolling ball represents the sloshing coffee acting back onto the cup and hand. The dynamics of this underactuated object is complex and may evolve into chaos, i.e., behavior becomes unpredictable even in the purely deterministic system [20,21]. Two previous studies investigating continuous interactions with this object showed that subjects seek to make the interaction dynamics predictable, even at the expense of exerting higher forces [19,22]. To simplify the demands on internal knowledge and prediction, another study explored how dynamic primitives may be used for transporting a cup to an endpoint where internal ball oscillations are minimized [23]. Bazzi and Sternad demonstrated in a point-to-point translation that subjects sought to attain stability, evaluated by contraction analysis [24–26]. Establishing predictability through stability is reasonable, as predicting and reacting to interaction forces in real time is extremely difficult due to long delays, ubiquitous noise, and limited bandwidth in the human sensorimotor system [27–29]. In such scenarios, continuous feedback-based control strategies, including optimal feedback control, may be challenged and the demands on predictive computations are high. Therefore, dynamic stability is beneficial as small perturbations or errors need not be explicitly corrected as the system returns to the attractor by itself. Common to all these studies is that subjects seek to make the interactions more predictable.

In all previous studies, subjects started the movement task with both the cup and the ball at rest. This significantly constrained the cup-and-ball dynamics during the initial part of the trial and, when moving the cup-and-ball continuously, subjects often required a significant amount of time to reach a steady state with predictable cup and ball dynamics. In daily behavior it is not uncommon that humans ‘initialize’ their objects to prepare for a task, especially when the object dynamics are unknown. Humans may first explore the internal dynamics by ‘jiggling’ the object prior to starting the intended movements. Such preparation of an object can be observed in many athletic routines, such as bouncing the ball before a tennis serve or preparing a diving board before launching the dive [30]. This study investigated how subjects explore and prepare the object prior to executing the target movement.

In general, a dynamical system starts from initial conditions and passes through a transient state before reaching a steady state. The duration of the transient depends on the initial conditions and some responses may never settle and result in chaotic behavior [31–33]. If given the chance to prepare an object’s dynamics prior to a task, can subjects find initial conditions that generate ‘simpler’ dynamics, evident in shorter transients and a higher degree of dynamic stability? These two aspects would preempt potentially chaotic behavior and enhance predictability. This study pursued the overall hypothesis that preparatory actions simplify the subsequent dynamic interactions and make them predictable.

Subjects were instructed to move a cup with a ball rolling inside and to settle into a sufficiently fast and challenging back-and-forth movement paced by a metronome. At the beginning of each trial, subjects could freely explore the dynamics of the object by “jiggling” the cup and ball to find their preferred initial conditions to prepare them for the subsequent rhythmic movements. We hypothesized that subjects seek to improve predictability in the hand-cup-ball interactions. As predictability is a broad concept, we separately examined the chosen initial conditions, the duration of the transient in the 15-s trial, and the stability of the steady state portion of the trial. We tested the following three specific hypotheses: *Hypothesis 1*: All subjects converge to a subset of initial conditions to make the ensuing dynamics more predictable. Two alternatives are possible: a) all subjects converge to the same subset of initial conditions afforded by the object; b) subjects find their individually preferred initial conditions. *Hypothesis 2*: These preferred initial conditions lead to shorter transients that reach a predictable steady state earlier. *Hypothesis 3*: The steady state exhibits dynamic stability as this affords robustness to small perturbations obviating error corrections. To challenge these expectations, three alternative hypotheses were formulated that were derived from extant computational studies on motor control: minimization of expended effort [14,34–37], maximization of smoothness [13,15], and reduction of risk [38,39].

The experimental results supported the overall hypothesis that subjects indeed increased predictability of their cup and ball dynamics. Subjects converged to initial conditions, transients became shorter, and the stability of the steady state increased with practice. In contrast, none of the alternative hypotheses found support by the data. To further scrutinize these experimental results, simulations of the cup-and-ball system examined these findings more specifically. Using inverse dynamic simulations, we identified the subset of initial conditions that led to more predictable interaction forces. Forward dynamic simulations with a first-order impedance controller generated solution spaces for these hypothesized objectives that could be compared with human data. Results showed that subjects converged to the manifold of initial ball angles that achieved shorter transients, enabling task performers to reach a stable steady state faster. Surprisingly, this strategy was more effortful and less smooth, unlike the observed behavior in simple reaching movements. In addition, subjects increased the ‘riskiness’ of interactions over their trials. This work presents first evidence that, given the opportunity, subjects learn to prepare the object to simplify subsequent complex object interactions and make them stable and predictable.

## Results

### Experimental task

**Fig 1:**
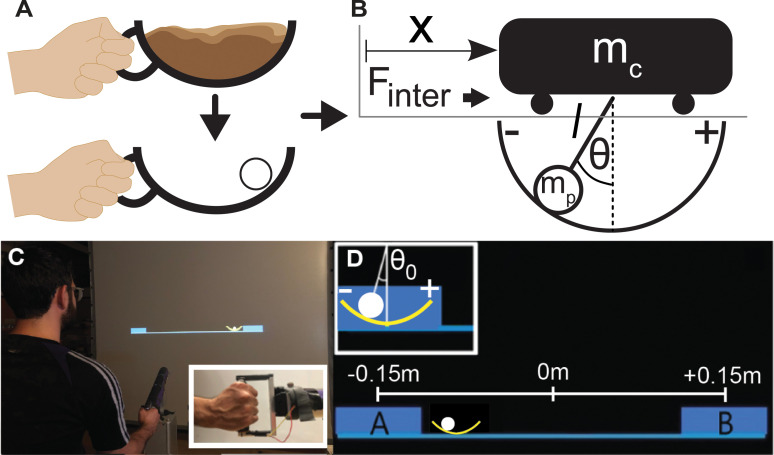
**Task design and experimental setup**: **A.** The task involved moving a two-dimensional cup with a ball sliding inside, inspired by humans carrying a cup of coffee. **B.** Mechanical model of a cart and pendulum. If the ball is at the bottom of the cup, the ball angle is defined as zero with positive and negative directions as shown. Hand movements that displace the cup on the horizontal line create interaction forces, *F_inter_*. **C.** The virtual experiment: A participant stands in front of the large screen and holds the handle of the HapticMaster robot to move the cup in the virtual environment. The inset shows the subject’s grip on the robot handle. **D.** Two rectangles on the screen delimited the cup movement amplitude, although they were larger than the cup allowing for some tolerance to downplay accuracy demands. Box A and Box B were 0.30 m apart, located at -0.15 m and 0.15 m, respectively.

The task of transporting a ‘cup of coffee’ was simplified to moving a two-dimensional arc with a ball ‘rolling’ inside (Fig 1A and 1B). The system was mechanically equivalent to a suspended frictionless pendulum attached to a cart moving on a frictionless surface. The ball acted as the pendulum bob and the cup position corresponded to the cart position; the cup itself was the semicircular arc that the pendulum bob traversed. The model and its equations of motion are detailed in the methods. Whenever the ball angle exceeded ±50 deg it would visually ‘spill’ from the cup, resulting in a failed trial.

The cup-and-ball system was implemented in a virtual environment, where subjects moved the cup via a haptic manipulandum (Fig 1C). At the beginning of each trial, the cup was positioned in Box A with the ball resting at the bottom of the cup (θ = 0 deg, Fig 1D). Subjects were instructed to move the cup rhythmically between Box A and Box B, following a metronome without losing the ball. The rim height was 50 deg, so the ball angle had to satisfy: |θ| < 50 deg (Fig 1D). The centers of the two rectangles were at -0.15 and 0.15 m, respectively, defining the cup amplitude as 0.30 m. Prior to starting each trial, subjects were encouraged to explore the cup and ball dynamics by ‘jiggling’ the cup back and forth to best prepare for the following continuously rhythmic movements. These preparatory movements had no time limit and were self-paced, i.e., there was no metronome. Once subjects felt ready, they moved the cup towards the right. Upon entry into Box B, the metronome started, and subjects continued to move rhythmically, paced at 0.60 Hz for 15 s. The last zero-crossing of cup velocity before starting the prescribed rhythmic movement marked the end of the preparation interval and was the moment where initial conditions were determined. Rhythmic movements were chosen to create continuous movements over some duration on a limited screen. The frequency of 0.60 Hz was chosen as it is close to the natural frequency of the pendulum (0.74 Hz) that defines the anti-resonance frequency of the cup-and-ball system. Maintaining cup movements close to this frequency is challenging, as shown previously [40].

**Fig 2:**
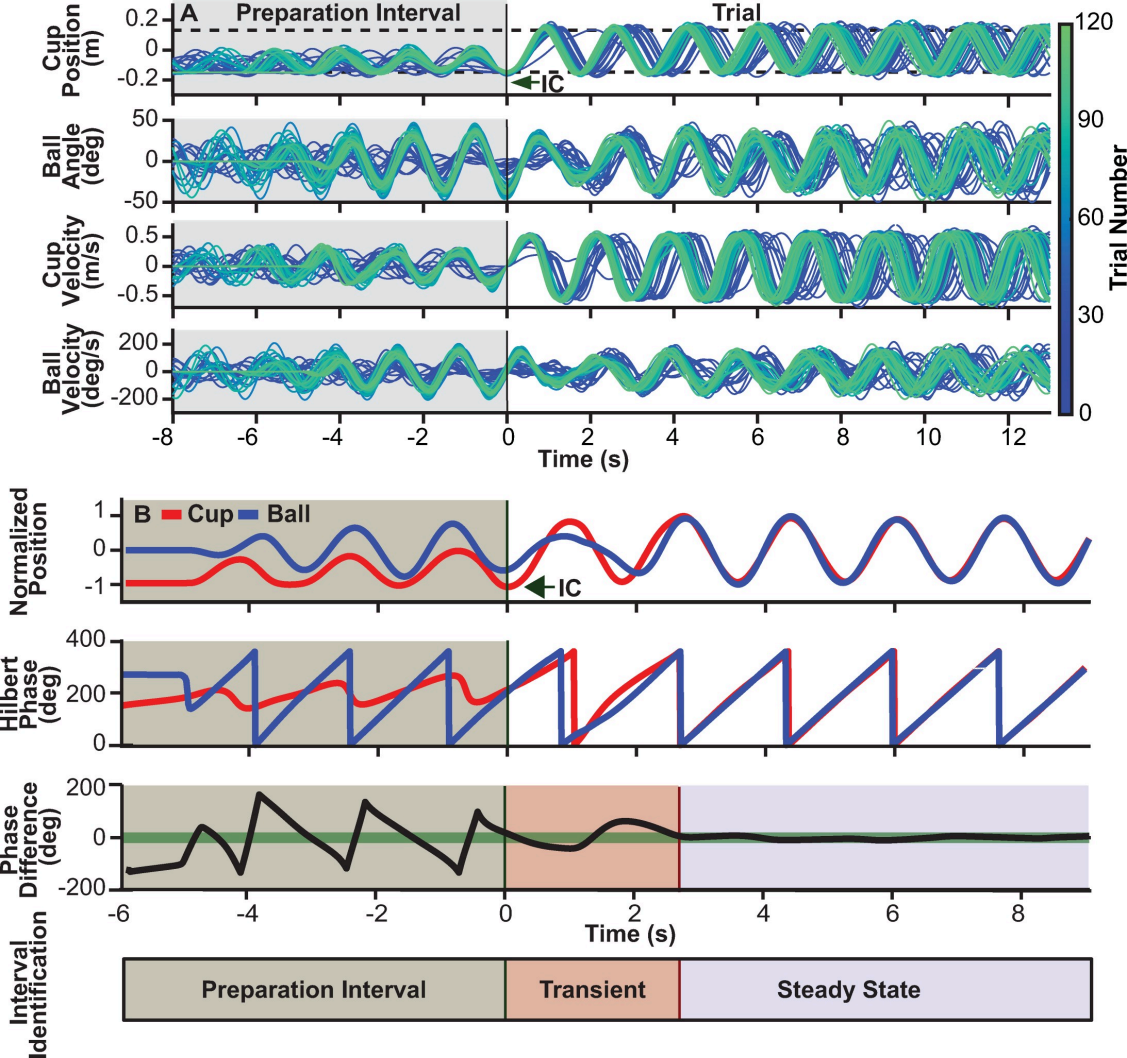
Object kinematics and transient calculation. **A.** Kinematics of the cup and ball of one sample subject: cup position, ball angle, cup velocity, and ball velocity. The panels show 60 trials (every other trial, with the sequence indicated by the color gradient). The dashed black lines in the top panel with cup position show the instructed cup amplitude, defined by the centers of Box A (-0.15m) and Box B (0.15m). The time series were aligned at the moment of trial initiation when the cup velocity was at its last zero prior to reaching Box B for the first time and starting the regular rhythmic movement. Initial conditions (IC) of all four variables were determined at this time point. **B.** Top panel shows cup position normalized to ±1 and ball angle similarly normalized between ±1. Calculation of relative phase between cup and ball trajectories to parse each trial into a transient and a steady state portion. To demarcate the end of the transient and start of the steady state, the instantaneous phase differences between cup and ball position were computed for the entire trial using the Hilbert Transform. The time point when the phase difference entered and remained within a threshold shown by the thick green line (27 deg or ±15% of the maximum phase difference of 180 deg) defined the end of the transient and start of the steady state.

Fig 2 illustrates the trajectories of cup and ball of a single subject, how initial conditions were determined, and how the trials were segmented into preparation interval, transients, and steady state. Fig 2A shows 60 out of 120 trials (every other trial) overlaid and aligned at the last zero velocity before moving towards Box B; lighter green indicates later trials. Fig 2B shows cup and ball trajectories of a single trial over a shorter segment to highlight how cup and ball reached a steady state with in-phase oscillations. To determine the end of the transient, relative phase between cup and ball was calculated, based on continuous Hilbert phase for each trajectory. A threshold of |27| deg in relative phase (±15% of maximum phase difference 180 deg) determined the end of the transient and the beginning of steady state. The standard deviations of relative phase served as indicator for stability during the steady state.

### Subject performance

**Fig 3:**
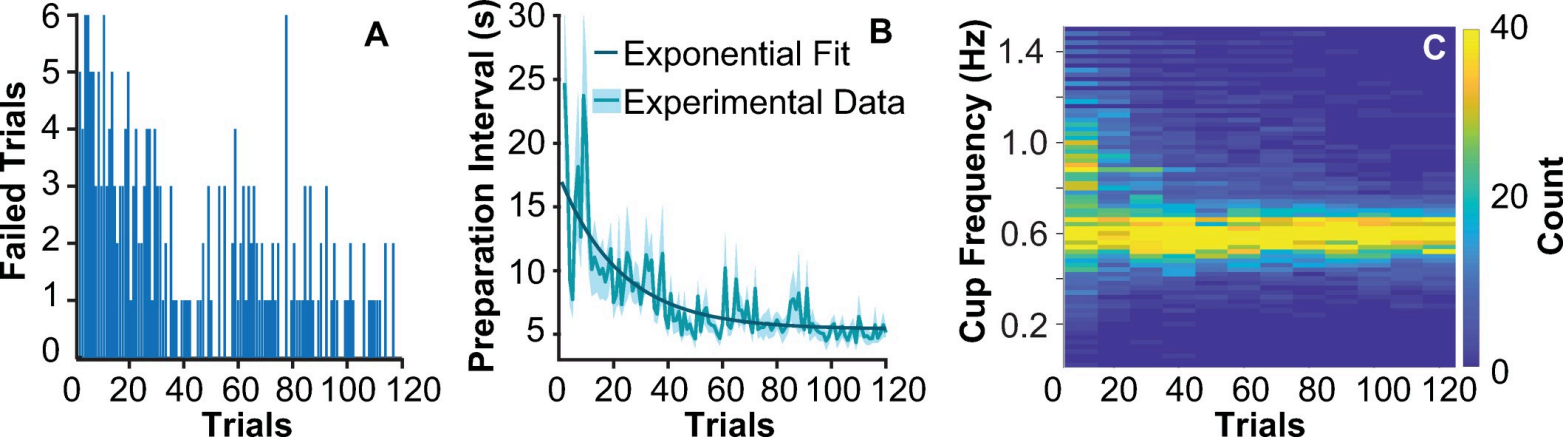
**Subject performance: A.** Number of subjects that lost the ball for each trial number. **B.** Duration of the preparation interval averaged across subjects over trials. The fluctuating solid line represents the mean, the shaded area represents the standard error across 11 subjects. The dark solid lines represent the exponential fit to the data, $A(1+e^{-\frac{t}{\tau }})+B$. The fitted parameters were: *A* = 12.75, *τ* = 22.48, *B* = -7.41, *R^2^* = 0.60 (see methods and statistical analyses). **C.** Frequencies of cup cycles for all subjects during the preparation interval, plotted in bins of 10 trials. The color gradient denotes the number of cycles in each frequency bin; yellow shows high numbers.

Overall, subjects followed the task instructions and after initial exploration and preparation of the object, they managed to synchronize their cup movements to the metronome pace. However, the task was difficult and in 22% of all trials (335 trials out of 1560 trials for all 13 subjects) the ball angle exceeded the rim angle at 50 deg and ‘escaped’. Two of these 13 subjects lost the ball in 55 and 60 trials, respectively and were excluded as outliers from all further analysis [41]. The percentage of failed trials across the remaining 11 subjects was 16.71%, i.e., 220 trials out of 1320. Of these failed trials, 18 trials were aborted during the preparation interval, while 202 trials failed predominantly in the first third of the experiment as the histogram in Fig 3A shows. The failed trials of the 11 subjects were excluded from subsequent analysis.

Given that the metronome frequency was set to 0.60 Hz, the average overall frequency at which subjects oscillated the cup during the entire trial was 0.58 Hz (range: 0.55–0.63 Hz). While the average frequency slightly increased from the first 5 to the last 5 trials (from 0.564 ± 0.033 to 0.588 ± 0.012 Hz, t(10) = -3.10, p = 0.0112), the frequencies in the last 30 trials did not significantly differ from 0.60 Hz, t(10) = 1.16, p = 0.2741. The average peak-to-peak cup amplitude increased from the initial 5 trials to the last 5 trials (from 0.298 ± 0.012 m to 0.309 ± 0.011 m, t(10) = -3.71, p = 0.0041). The cup amplitude at the end in the last 30 trials did not differ from 0.30 m, t(10) = -0.81, p = 0.4347. Hence, overall subjects satisfied the task instructions.

The time that subjects used to explore and prepare the cup-and-ball for the subsequent movements was unrestricted. However, over the course of the experimental session, this preparation interval decreased showing that subjects needed less time to prepare for their movements in later trials. Fig 3B shows the average times with their standard errors across subjects for each of the 120 trials. The data were fit by an exponential function with a time constant of 22.48 trials and R^2^ = 0.60. Note that for many trials, especially in the early part of the experiment, fewer than 11 successful values were available; the standard error was calculated over the available number of subjects per trial. The preparation interval decreased from 15.35 ± 9.81 s in the first 5 trials to 5.24 ± 2.07 s in the last 5 trials (paired *t*-tests: t(10) = 3.45, p = 0.0062). To further visualize how subjects prepared the cup-and-ball system, Fig 3C displays a histogram of the individual cup movement cycles for all subjects during the preparation interval. The frequencies of all cup cycles were binned into intervals of 10 trials; the yellow colors denote frequencies that were employed more frequently. The histogram shows that during initial trials, subjects explored a wider range of cup cycle frequencies and then converged to moving the cup at the prescribed frequency of 0.60 Hz prior to initiating the regular rhythmic task.

### Predictability of cup and ball dynamics

**Fig 4:**
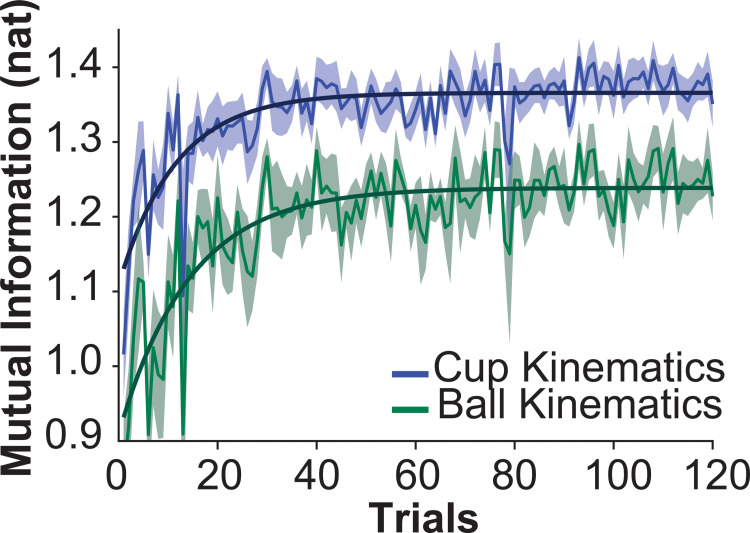
**Predictability of cup and ball dynamics measured via mutual information**: Mutual information between *F_inter_(t)* and cup kinematics averaged across subjects over trials is shown in blue. Parameters of the exponential fit were: *A* = 0.26, *τ* = 11.54, *B* = 1.11, and *R*^2^ = 0.65. Mutual information between *F_inter_(t)* and ball kinematics averaged across subjects over trials is shown in green. Parameters of the exponential fit were: *A* = 0.33, *τ* = 14.14, *B* = 0.91, and *R*^2^ = 0.71.

The *overall hypothesis* was that subjects seek to increase the predictability of the interactive dynamics between the applied force and the object. Consistent with previous work [18,19,22], mutual information (*MI*) between the interaction force *F_inter_(t)* and the object dynamics was used to quantify predictability. Here, we computed *MI* between *F_inter_(t)* and the cup phase and the ball phase separately; both metrics were computed over the entire trial duration including transient and steady state. Fig 4 shows that *MI* for both the cup and ball kinematics increased exponentially with a relatively short time constant of 11.54 trials (*R*^2^ = 0.65) before reaching a plateau. Comparing the first and last 5 successful trials shows that *MI* increased from 1.219 ± 0.122 nat to 1.371 ± 0.07 nat at the end, t(10) = -5.510, p = 2.581x10^-04^ (Fig 4). For reference, the maximum *MI* value between *F*_*inter*_(*t*) and the cup movement for the given parameters was 1.60 nat. *MI* between *F_inter_(t)* and the ball movement also increased with a time constant of 14.14 trials, *R*^2^ = 0.71. *MI* increased from 1.019 ± 0.187 nat in the first 5 trials to 1.245 ± 0.094 nat in the last 5 trials, t(10) = -4.866, p = 6.551x10^-04^. Both changes in *MI* were statistically significant, supporting the *overall hypothesis* that participants increased the predictability of their interactions with the cup-and-ball system.

### Hypothesis 1: Initial conditions

**Fig 5:**
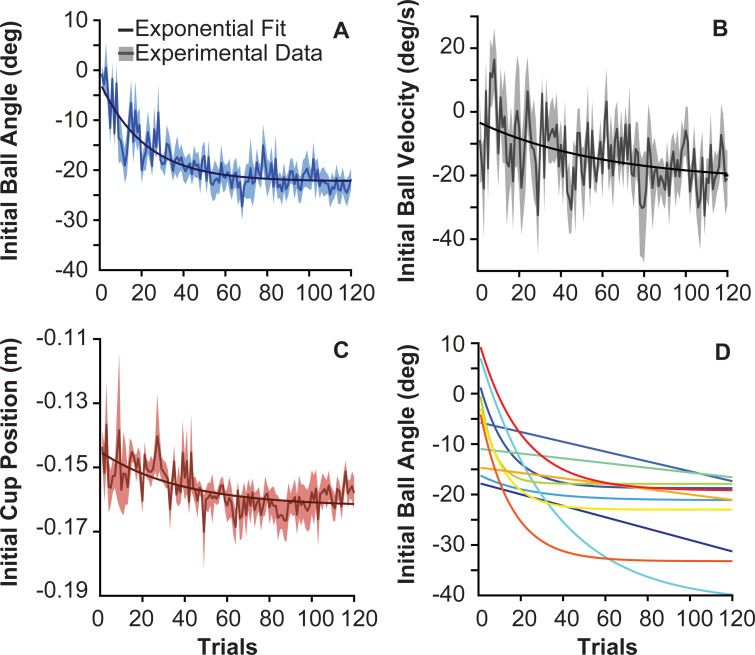
**Average initial conditions over trials: A.** Initial ball angle of 11 subjects across 120 trials. The solid blue line represents the mean, the shaded area represents the standard error across subjects (excluding their failed trials). The solid black line represents the exponential function fitted to the data: *A* = 19.86, *τ* = 21.82, *B* = -42.09, and *R^2^* = 0.75. **B.** Initial ball velocity; parameters of exponential fit: *A* = -14.59, *τ* = 58.38 and *B* = -2.86, and *R^2^* = 0.12. **C.** Initial cup position; parameters of exponential fit: *A* = 0.02, *τ* = 40.94, *B* = -0.18, and *R^2^* = 0.45. **D.** Exponential functions fitted to each individual subject’s initial ball angle over 120 trials.

Did subjects achieve this predictable behavior by preparing the cup-and-ball system appropriately and choosing favorable initial conditions? The initial conditions were ball angle *θ*_0_, ball angular velocity ${\dot{\theta }}_{0}$, and cup position *x*_0_; cup velocity ${\dot{x}}_{0}$ was 0 by definition. Fig 5A, 5B and 5C show the average and standard errors of all three variables across all subjects over the course of the 120 trials. Exponential fits over the plotted averages highlight the overall trends.

The initial ball angle *θ*_0_ changed from an average of -7.23 ± 7.43 deg across subjects in the first 5 successful trials to an average of -22.75 ± 7.31 deg in the last 5 successful trials, t(10) = 5.43, p = 2.883x10^-04^ (Fig 5A). The exponential fit across trials had a decay constant of 21.82 trials with *R*^2^ = 0.75. The initial ball velocity ${\dot{\theta }}_{0}$ across subjects changed only slightly from an average of -4.67 ± 19.26 deg/s in the first 5 trials to -11.74 ± 24.17 deg/s in the last 5 trials, t(10) = 0.85, p = 0.4160 (Fig 5B). The exponential fit to the data had a much longer decay constant of 58.38 trials and a low *R*^2^ = 0.12 due to the high variability. The initial cup position *x*_0_ across subjects started with an average of -0.152 ± 0.020 m in the first 5 trials and decreased to an average of -0.156 ± 0.013 for the last 5 trials, t(10) = 0.70, p = 0.4990 (Fig 5C). For reference, the center of Box A was located at -0.15 m, thus the average initial cup position was close to the center of Box A (Fig 1). The exponential fit to the data had a decay constant of 40.94 trials and a *R*^2^ = 0.45. These results on ball angle and velocity gave first support for *hypothesis 1* stating that, if given a choice, subjects converge to a subset of initial conditions. The variable that showed the most pronounced convergence towards preferred initial conditions was the initial ball angle. The starting position of the cup played a subordinate role.

The related important question was whether all subjects converged to similar initial ball states *θ*_0_ and ${\dot{\theta }}_{0}$, as opposed to finding their individually preferred initial conditions. Were the initial conditions determined by the object or was there a choice? To evaluate this question, each individual subject’s initial ball angles over the 120 trials were fitted by an exponential function to determine the asymptote, the *R^2^* of the fits ranged from 0.31 to 0.80. Fig 5D shows the exponential fits to each individual subject’s initial ball angles over trials. The asymptotes of the 11 subjects ranged from -17.87 to -41.51 deg, shown in Fig 5D. Comparing the last 5 trials of the 11 subjects with a one-way ANOVA identified significant differences, p = 6.1414x10^-14^. This result supports *hypothesis 1b* that each subject finds their own preferred initial condition and will be revisited below.

### Hypothesis 2: Transient duration

**Fig 6:**
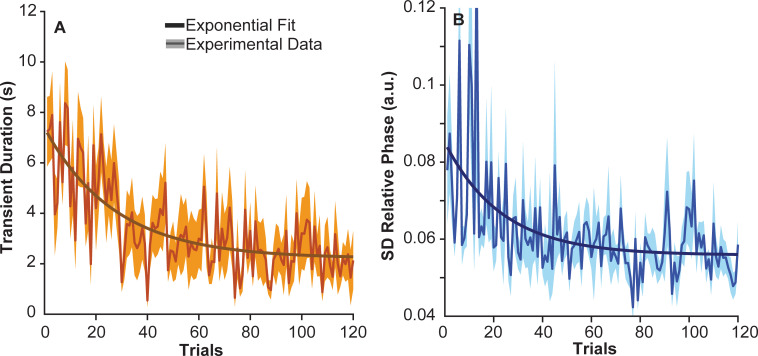
**Transient duration and stability of the steady state: A.** Transient durations of 11 subjects across 120 trials. The solid line represents the mean, the shaded area around this line represents the standard error across subjects. The dark solid line represents the exponential function fitted to the data: *A* = 5.174, *τ* = 27.29 and *B* = -2.963, and *R*^2^ = 0.56. **B.** Standard deviations of the relative phase between ball and cup position across subjects during the steady state interval. The data was fit with the parameters: *A* = 0.03, *τ* = 23.43 and *B* = 0.03, and *R*^2^ = 0.31.

*Hypothesis 2* stated that transients should become shorter as, by definition, transients imply non-stationary dynamics that is harder to predict. Note that subjects were only asked to move the cup rhythmically at the metronome pace to the best of their abilities; they were never explicitly told to decrease their transient duration or to reach a steady state. Fig 6A summarizes the change in transient duration across the 120 trials. The average transient across all subjects significantly changed with an exponential decline (*R*^2^ = 0.56); the decay constant *τ* was 27.29 trials. In the first 5 successful trials the average duration was 6.068 ± 3.035 s and decreased to 2.172 ± 1.952 s by the last 5 successful trials, *t*(10) = 3.58, p = 0.0019. This result supports *hypothesis 2*, showing that subjects shortened their transient and achieved a predictable steady state sooner.

### Hypothesis 3: Stability of steady state

*Hypothesis 3* stated that stability during steady state should increase, as predictability is also enhanced in a system that is stable. Steady state behavior in this coupled cup-and-ball system consists of two approximately sinusoidal movements that are either approximately in-phase or anti-phase. However, for the given cup movement frequency, cup and ball are close to in-phase, as evident from linear frequency response analysis (for the lossless system, they are exactly in-phase, but with hand impedance present, deviations arise) [20,40]. To assess the degree of stability in coupled oscillations, relative phase and its fluctuations have been used as a proxy [42,43]. Fig 6B show the standard deviations of relative phase during the steady state portion of the trials. The mean across subjects noticeably decreased over 120 trials, highlighted by the decaying exponential with a time constant *τ* of 23.43 trials (*R*^2^ = 0.31). In the first 5 trials the average was 0.074 ± 0.030 arbitrary units (a.u.) that significantly dropped to 0.050 ± 0.007 a.u. in the last 5 successful trials, *t*(10) = 2.61, p = 0.0166.

### Alternative Hypothesis 1: Interaction force or effort

**Fig 7:**
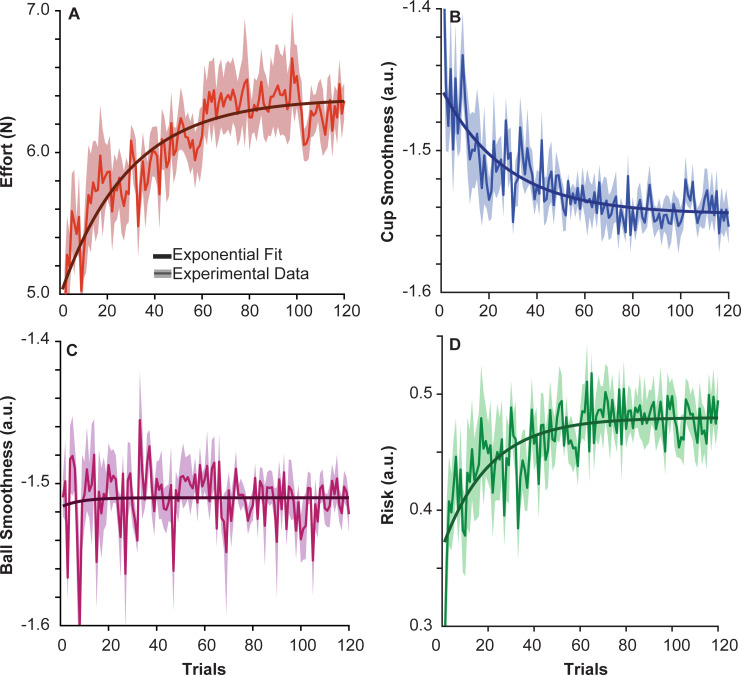
**Subject performance measured by effort, smoothness and risk metrics: A.** Root mean square (RMS) of *F_inter_*, a measure of effort, across subjects and trials. The solid red line shows the mean across subjects; the shading shows the standard error of the mean. The trend in the data was fit with: *A* = 1.396, *τ* = 28.76, *B* = 4.987, and *R*^2^ = 0.80. **B.** Smoothness of the cup trajectory quantified by the SPARC method averaged across subjects over 120 trials. The trend in the data was fit with: *A* = 0.108, *τ* = 19.44, *B* = -1.650, and *R*^2^ = 0.71. Note that larger negative numbers indicate less smooth kinematics **C.** Smoothness of the ball kinematics calculated using the SPARC method averaged across subjects over the experiment is shown in magenta. The trend in the data was fit with: *A* = -0.007, *τ* = 8.44, *B* = -1.5, and *R*^2^ = 0.003. **D.** Risk quantified as (1-energy margin); decreasing energy margin is expressed as increasing risk. The average risk across subjects over trials is shown by the solid line. The data was fit with the same exponential function: *A* = 0.086, *τ* = 23.32, *B* = 0.295, and *R*^2^ = 0.46.

The first *alternative hypothesis* was derived from several studies that showed that humans minimized effort during unconstrained movements [34,44,45]. The interaction force of the hand on the cup, *F*_*inter*_, was measured by a force sensor at the robot handle. The root mean square (RMS) of *F*_*inter*_ over the entire trial duration was used to quantify the exerted effort. The average RMS of *F*_*inter*_ or effort across subjects over trials is plotted in Fig 7A. Contrary to expectation, the exerted force onto the cup-and-ball increased across trials; the time constant of the exponential fit was 28.76 trials (*R*^2^ = 0.80). The average value in the first 5 successful trials was 5.240 ± 0.668 N and increased in the last 5 trials 6.327 ± 0.496 N, t(10) = -4.33, p = 3.2264x10^-04^. These results rejected *alternative hypothesis 1*, demonstrating that when interacting with a complex object subjects may even increase their effort due to other priorities.

### Alternative Hypothesis 2: Smoothness of cup and ball trajectories

Smoothness of the cup trajectory was quantified using the Spectral Arc method (SPARC) that was specifically developed to assess smoothness in continuous or rhythmic kinematics (see methods Eqs 7–9 [46,47]). The average smoothness of cup kinematics across subjects per trial decreased throughout the experiment, i.e., cup trajectories became less smooth (Fig 7B). The values decreased from -1.461 ± 0.083 a.u. in the first 5 successful trials to -1.549 ± 0.037 a.u. in the last 5 trials, t(10) = 3.19, p = 0.0046. The exponential fit had a decay constant of 19.44 trials (*R*^2^ = 0.71). Fig 7C shows the corresponding smoothness of the ball kinematics across subjects over 120 trials. Ball smoothness showed no significant trend over the course of the experiment, evidenced by the insufficient fit of the exponential function (*R*^2^ = 0.0033). The values were -1.516 ± 0.041 a.u. in the first 5 successful trials and -1.513 ± 0.025 a.u. in the last 5 successful trials, t(10) = -0.16, p = 0.8753. These results demonstrate that in continuous rhythmic interaction with an object with nonlinear internal dynamics, smoothness of the object dynamics is not prioritized, providing evidence against *alternative hypothesis 2*.

### Alternative Hypothesis 3: Energy margin or risk

To assess if subjects minimized the likelihood of losing the ball, an energy margin was calculated that quantified the difference between the energy of the ball in its current state and the minimum energy leading to ball escape (see methods Eqs 10–15). Smaller energy margins signify higher risk of the ball angle exceeding the height of the cup and escaping. The energy margin was transformed to 1-energy margin to obtain an intuitive metric for the risk of failure. Risk is a normalized and unitless value between 0 (ball resting at the bottom of the cup) and 1 (ball at the verge of escaping the cup). Fig 7D shows that risk averaged across subjects increased exponentially with a time constant of 23.32 trials (*R*^2^ = 0.46). The values in the first 5 trials, 0.405 ± 0.087 a.u. increased to 0.487 ± 0.064 a.u. in the last 5 trials, t(10) = -2.52, p = 0.02. This result provides evidence against *alternative hypothesis 3*. Despite the instruction to avoid losing the ball from the cup, subjects increased the likelihood of failure throughout the experiment, potentially prioritizing other objectives over risk.

### Simulation results and hypothesis testing

To further scrutinize subjects’ behavior and their choice of initial conditions, inverse dynamics simulations were performed. Setting sinusoidal cup trajectories as the desired output, the calculations identified the necessary input, i.e., interaction forces for all feasible initial ball states. The computed interaction forces gave insight into which initial conditions afforded the least complex forces to achieve rhythmic cup trajectories. To also evaluate whether the initial conditions supported any of the hypothesized objectives, forward dynamics simulations used a simple control model to generate cup and ball kinematics. Sweeping through an array of initial conditions, the generated trajectories were analyzed in the same way as the experimental data. The results created solution spaces in which the data could test the hypotheses about predictability and possible alternatives.

#### Inverse dynamics: Effect of initial ball angle on interaction forces

**Fig 8:**
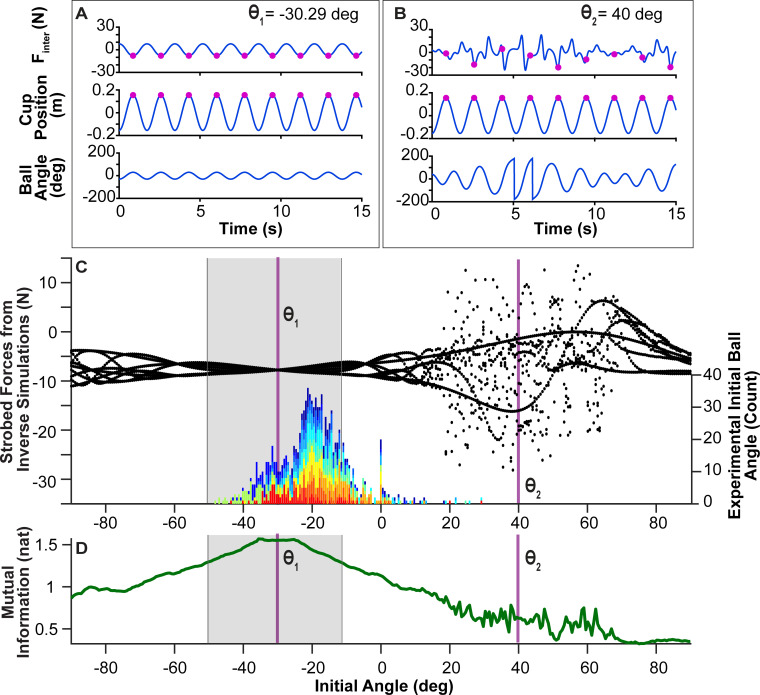
**Effect of initial ball angle on interaction forces: A.** Simulated interaction force *F_inter_(t)*, cup position *x(t)*, and ball angle *θ*(t), for rhythmic cup displacement starting with *θ*_1_ = −30.29 deg and ${\dot{\theta }}_{0}$ = −15.81 deg/s, the average initial ball velocity at the end of the experiment across all subjects. The cup frequency and amplitude were set to experimental averages 0.58 Hz and 0.308 m (peak-to-peak). **B.** Simulated interaction force *F_inter_(t)*, cup position *x(t)*, and ball angle *θ*(t), for a trial initiated with *θ*_2_ = 40 deg. The initial values for ${\dot{\theta }}_{0}$, cup frequency and amplitude were the same as in panel A. **C.** Distribution of strobed force *F_inter_(t)* for all initial ball angles: At every maximum of *x(t)*, the value of *F_inter_(t)* was determined, as displayed by the magenta points at each peak of *x(t)*. The marginal distributions of the strobed force profiles are plotted against the respective initial ball angle *θ*_0_ between -90 deg and +90 deg (the step size was 5 deg). *θ*_1_ and *θ*_2_ are marked with vertical lines. The gray box overlaid onto the figure denotes the range of initial ball angles for which the simulated ball position data remained below ± 50 deg, i.e. the ball did not escape. The figure also includes a histogram of experimental values of *θ*_0_ for all trials pooled over all subjects; different subjects appear in different colors. The mode of the distribution of the experimental data was at -22.68 deg. **D.** Mutual information (*MI*) between the simulated *F_inter_(t)* profile and the cup kinematics for each *θ*_0_. Higher *MI* values indicates higher degrees of predictability. The initial ball angles that produce the highest *MI* values align with the *θ*_0_ that produce the least complex distributions of the strobed *F*_*inter*_(*t*).

Inverse dynamics calculations were performed to evaluate the effect of initial conditions on the interaction force *F*_*inter*_ required to generate the desired rhythmic movement of the cup. These calculations did not assume any controller, but instead computed the temporal profile of *F*_*inter*_ needed to generate a sinusoidal cup trajectory for specific initial ball angles. The simulations focused on the effect of *θ*_0_ on *F*_*inter*_ as the experimental data showed the clearest convergence in initial ball angles. The desired cup displacement was modeled as a sinusoid. To afford the best comparison to the experimental data, the sinusoidal cup frequency was set to the subjects’ mean frequency 0.58 Hz, and the peak-to-peak amplitude was set to the subjects’ mean amplitude 0.308 m. The initial conditions for the cup were set to: *x*_0_ = -0.15 m, $\dot{x_{0}}$ = 0 m/s, and initial ball velocity $\dot{\theta_{0}}$ was set to -15.81 deg/s, which was the average $\dot{\theta_{0}}$ across all subjects. While these values were kept constant, the inverse calculations swept through all values of *θ*_0_ between -90 deg and 90 deg, with a step size of 5 deg. Fig 8A and 8B display two profiles of *F*_*inter*_(*t*) (top panels) that generated the same sinusoidal cup displacement *x*(*t*), but the simulations were initialized with different initial ball angles: *θ*_1_ = −30.29 deg on the left (Fig 8A), and *θ*_2_ = 40 deg on the right (Fig 8B).

The first profile *F_inter_(t)* produced a consistent periodic and predictable force profile. The second profile *F_inter_(t)* shows highly irregular force values that ranged between 20 N and −20 N.

To summarize the dependence of *F*_*inter*_ profiles on the initial ball angles *θ*_0_, stroboscopic plots were produced. For each simulation run with a given *θ*_0_, *F*_*inter*_(*t*) was strobed at the maxima of the cup position trajectory; Fig 8A and 8B show these strobed values as magenta points in the force profiles. The marginal distributions of these strobed *F*_*inter*_ values, shown at the *y*-axis, were plotted against the respective *θ*_0_ value that had generated that force profile (Fig 8C). The figure shows these marginal distributions by black points; *θ*_1_ and *θ*_2_ are highlighted with vertical magenta lines. The initial ball angle *θ*_1_ that produced the simplest *F*_*inter*_ profile was at −30.29 deg; the value of *F*_*inter*_ at the maxima of the cup was always -7.94 N. Note that these simulations included all generated time series of *F*_*inter*_(*t*), even if the ball exceeded the rim angle of the cup (50 deg). Hence, the gray-shaded box overlaid on the figure marks those initial ball angles for which the simulated ball positions did not exceed ± 50 deg, i.e., did not escape the cup. While there is one optimum, leading to a strictly periodic solution, the range of possible initial conditions was from -50 to -11.51 deg. Interestingly, the summary plot of strobed force values has remarkable similarity with bifurcation diagrams that show the period-doubling route to chaos [48,49]. Hence, Fig 8C indicates that for certain initial conditions this seemingly simple cup-and-ball system can indeed display chaos [31].

To also present a single value that captures the complexity of the interaction force profile, *MI* between the cup kinematics and the simulated *F*_*inter*_(*t*) for each *θ*_0_ was calculated. Higher *MI* indicates higher predictability between the interaction forces and the cup kinematics. Fig 8D shows that the initial ball angles that produced the highest *MI* values align with those *θ*_0_ that produce the least complex distributions of strobed *F*_*inter*_(*t*) values.

To compare these simulation results to the experimental data, a histogram of *θ*_0_ for all trials from all subjects was inserted into the diagram (Fig 8C); different subjects are distinguished by different colors. The distribution is broad with one primary mode and a second less pronounced mode. The primary mode of the distribution was at -22.68 deg, which was close to, but not exactly at *θ*_1_ = -30.29 deg, the angle that generated the simplest force profile. A comparison of the last 30 trials showed that the mean values across all subjects (-22.13 ± 6.95 deg) did not significantly differ from *θ*_1_, t(10) = -1.13, p = 0.2867. These results provide further support for *hypothesis 1* that subjects seek initial conditions that prepare them for predictable interaction dynamics.

The Additional Analysis section includes a parallel set of inverse dynamics simulations performed with initial ball velocity on the x-axis, and different fixed initial ball angles. Similar strobed force diagrams were obtained showing that the experimental initial ball velocities align with those that produce more regular interaction forces, although the data were more variable.

#### Forward simulations with an impedance controller

To gain more insight into the control objectives and test the specific hypotheses about predictability and the alternative hypotheses, forward simulations were conducted for the range of possible initial ball conditions. To avoid making too many assumptions about the controller, a simple first-order controller with mechanical impedance was used to drive the cup.


$$F_{inter}=-K[x-x_{des}]-B[\dot{x}-{\dot{x}}_{des}]$$ (1)

The desired cup movements *x*_*des*_ and ${\dot{x}}_{des}$ were assumed to be a sinusoid to approximate the instructed cup movement. To conduct the simulations, first the impedance parameters, stiffness *K* and damping *B*, were estimated for each trial of each subject. Using an optimization procedure, described in the methods, the modes of the impedance estimates across all trials and subjects were determined: *K* = 40 N/m and *B* = 40 Ns/m [22]. Using these as representative values, forward simulations were run to assess how different initial ball angle and velocity values generated different transient durations, stability of steady state, effort, smoothness and risk. As above, the cup amplitude was set to 0.308 m and cup frequency was set to 0.58 Hz; the initial cup position *x*_0_ was -0.15 m, and cup velocity was ${\dot{x}}_{0}=0$. In the simulations the initial ball angle was varied from -50 deg to 45 deg with a step size of 5 deg; the initial ball velocity was varied from -160 deg/s to 160 deg/s with a step size of 5 deg/s.

#### Hypotheses 2 and 3: Transient duration and stability of steady state

**Fig 9:**
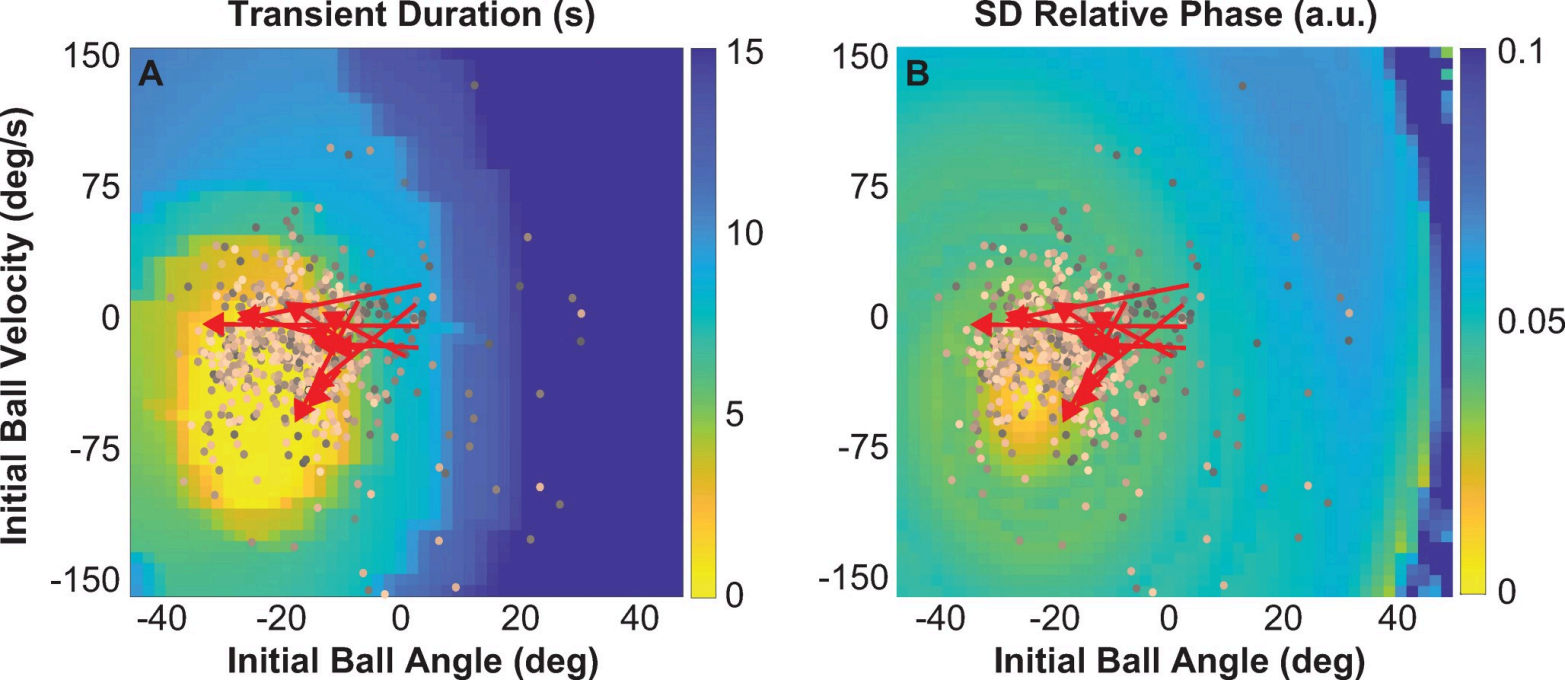
**Predicted effect of initial ball states on transient duration and stability of the steady state:** Simulated results for transient duration and variability of relative phase during steady state. The results were generated by forward simulations using an impedance controller for different initial ball angles and velocities; the impedance parameters were: *K* = 40 N/m and *B* = 40 Ns/m; cup frequency was fixed at 0.58 Hz. In the simulated maps experimental data of ball angles and velocities from all 1100 successful trials are overlaid; darker colors indicate early trials and lighter colors show later trials. For each subject, the means of the first 5 trials and the last 5 trials are connected and shown by red arrows. **A.** Simulated transient durations for different initial ball angles and velocities. **B.** Stability of steady state calculated as standard deviations of relative phase between cup and ball position.

Transient durations and relative phase fluctuations were calculated for each simulation run using the same methods as for the experimental data (Fig 2B). Fig 9A shows a heat map of the transient durations predicted by initial ball angles and initial ball velocities. The lighter colors identify the regions that result in relatively short transients; blue indicates longer transient durations. Note that there is a region or manifold of transient durations of 0 s that were generated by initial ball angles between -21.35 deg and -38.54 deg and initial ball velocities between 0.43 and -102.70 deg/s; the center of this yellow-shaded area was located at *θ*_0_ = -29.14 deg, $\dot{\theta_{0}}$ = -54.70 deg/s. To compare the simulated results with human data, all 1100 successful experimental trials from all subjects were overlaid on the heat maps based on their measured initial ball states. A color gradient in the data points indicates the progression from early (darker) to late trials (lighter). In addition, each individual subject’s change is shown by a red arrow that connects the mean of the first 5 with the last 5 trials. Both visualizations show a trend from small positive ball angles to more negative ball angles; 5 out of 11 subjects also displayed increasingly negative ball velocity. This overlay shows that although the final values differed across subjects, all arrows point towards the region with the shortest transients. This redundancy in the mapping between initial ball states and transient duration may explain why the individual subjects converged to different initial conditions (see Fig 5A).

Fig 9B shows the standard deviations of relative phase between the cup and ball position during the steady state interval using the same method as for the transient durations. In the heat map low variability or high stability is indicated in yellow, and high variability is shown in dark blue. The initial ball angle and ball velocity that achieve the highest degree of stability are -27.08 deg and -45.41 deg/s, respectively. The simulated map includes the same experimental data as in Fig 9A. Again, the arrows point towards the yellow areas, consistent with the location of the point of highest stability, in support of *hypothesis 3*. Note that the optimal regions for transient duration and standard deviations of relative phase overlap, but the optimum for stability is a point, while zero-duration transients form a manifold, showing redundancy in the choice of initial conditions.

#### Alternative hypotheses: Effort, smoothness and risk

**Fig 10:**
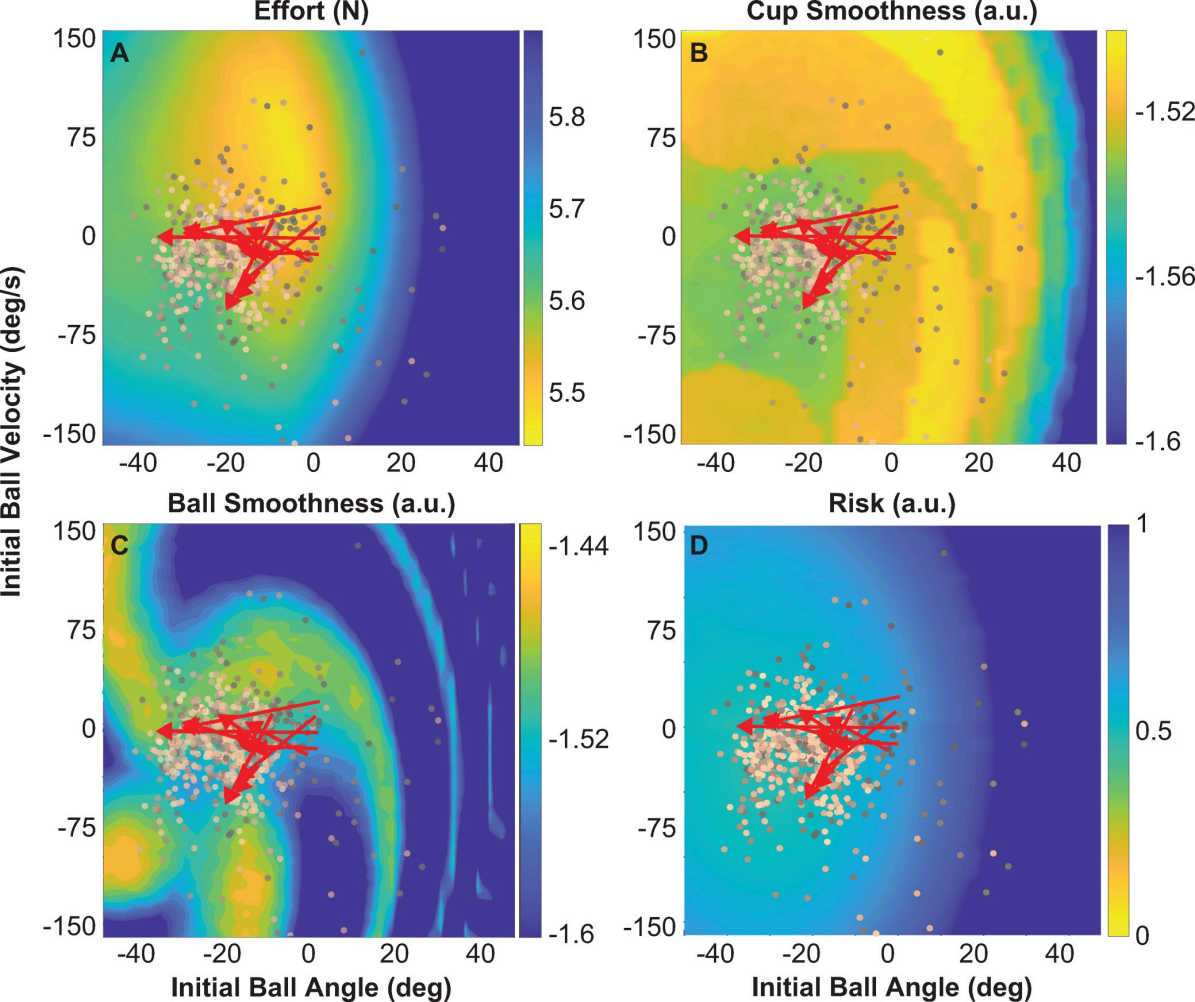
**Predicted effect of initial ball states on effort, smoothness and risk metrics:** Simulated metrics generated by forward simulations using an impedance controller for different initial ball angles and velocities. The impedance parameters were: *K* = 40 N/m and *B* = 40 Ns/m; the cup frequency was fixed at 0.58 Hz for all simulations. In all simulated maps experimental data of ball angles and velocities from all 1100 successful trials are overlaid; darker colors indicate early trials and lighter colors show later trials. For each subject, the means of the first 5 trials and the last 5 trials are connected and shown by red arrows. **A.** Effort expressed as RMS of *F*_*inter*_(*t*). **B.** Simulated cup smoothness values calculated for different initial ball angles and initial ball velocities. **C.** Simulated ball smoothness values calculated for different initial ball angles and initial ball velocities. **D.** Risk or the inverse of energy margins. In all simulations, yellow indicated the desired regions.

The same procedure was used to evaluate three alternative hypotheses. Effort exerted in each simulated trial was quantified using the RMS *F*_*inter*_(*t*). The effect of initial ball states on the resulting RMS force is displayed in Fig 10A. The yellow areas reveal initial ball states that minimized RMS force throughout the trial, whereas the dark blue areas mark initial ball states that would increase RMS force. The minimum simulated value of 5.48 N was produced by *θ*_0_ = -7.03 deg and $\dot{\theta_{0}}$ = 57.72 deg/s. Comparing these predicted regions with the corresponding experimental data clearly shows that subjects moved away from the optimal initial ball states that would reduce effort. These simulations are consistent with the experimental observations reported above (Fig 7A) and show that the chosen initial conditions can lead to these counter-intuitive results that reject *alternative hypothesis 1*.

Cup and ball smoothness were calculated using the SPARC metric [46,47] on the simulated cup velocity and ball velocity for each of the forward simulation run. Fig 10B and 10C show the solution spaces together with the same experimental data as before. The initial ball states that produced the smoothest cup trajectory value -1.499 a.u. were *θ*_0_ = -44.27 deg and $\dot{\theta_{0}}$ = 155.1 deg/s. The highest ball smoothness in simulation was -1.450 a.u. and was achieved with *θ*_0_ = -44.27 deg and $\dot{\theta_{0}}$ = -114.1 deg/s. The subjects’ changes in cup smoothness were opposite to the optimal regions predicted by the forward simulations: smoothness decreases, rejecting *alternative hypothesis 2*. While the individual subjects’ ball smoothness did not decrease as clearly, it also showed little convergence to the optimum predicted by the model simulations.

Risk in the simulated ball trajectories was quantified by 1-energy margin as above (Fig 10D). The minimum risk value was 0.49 a.u. obtained with *θ*_0_ = -32.81 deg and $\dot{\theta_{0}}$ = -28.22 deg/s. Yellow in the colormap shows initial ball states that produce kinematics with low risk, although the gradient is shallow. Of all the objectives, risk showed the least amount of variation based on initial ball states, visible in the limited color changes. Note also that this simulation was performed keeping the cup frequency fixed to the mean value. Even though the changes in cup frequency were small (see above), the solution space changed with frequency in a subtle manner. Computing correlations between cup frequency and risk showed a good correlation of 0.78. Subsequent correlations between cup frequency and risk showed that risk values depended on cup frequency, such that higher cup frequencies produced higher risk values. In sum, the analyses of risk were less informative.

## Discussion

This study examined strategies that humans adopted when moving a non-rigid object, a cup with a ball rolling inside, continuously between two targets. Due to its internal dynamics, seemingly simple cup movements could generate complex dynamic behavior that ranged from predictable to chaotic, depending on the initial ball states. In this experiment, subjects were given the opportunity to explore and prepare the cup-and-ball dynamics before starting their continuous cup movements that were paced by a metronome. The *overarching hypothesis* was that humans strive to make the interaction dynamics more predictable. The experimental results supported this overarching hypothesis, specified with support for the three specific hypotheses. As humans prepared the object for the upcoming task, they indeed converged to a subset of initial conditions, most prominently ball angles, but also ball velocities. While individuals differed in their choices, each subject chose a narrow subset of initial ball angles (*hypothesis 1*). The transient portions became shorter with practice such that subjects reached the steady state portions faster (*hypothesis 2*). Steady state performance implies predictable relations between interaction force and object dynamics; this stable relation can be characterized by fluctuations of relative phase between hand and ball. As expected, variability decreased with practice, i.e., the degree of stability of this steady state increased (*hypothesis 3*). Three alternative objectives (minimum effort, maximum smoothness, and minimum risk) could not account for interacting with a complex object. Given the widespread acceptance of these criteria in unconstrained movement tasks, our results demand a pause and further scrutiny. Results from inverse dynamics supported that the initial ball states that produced the most predictable cup-ball interactions in simulation coincided with those chosen by human subjects. Forward simulations with a first-order impedance control model based on estimated hand impedance revealed that the experimental initial conditions approximately matched the theoretical solutions that shortened transient durations and reached a stable steady state. Analogous simulations of *three alternative hypotheses*, effort, smoothness and risk, created very different solution spaces that did not match the experimental data.

In sum, the overarching hypothesis that human subjects aim to increase predictability of the interaction dynamics was supported.

### Preparing the object for the task: Sensitivity to initial conditions

Preparatory movements are frequently observed in sports and often become a characteristic signature of the athlete. For example in baseball, pitchers converge to specific ‘wind-up’ movements prior to throwing the baseball [50]. The gymnastic routines of an elite diver after take-off are largely dependent on their often idiosyncratic preparatory actions on the spring board [30,51,52]. In daily manipulation tasks, humans prepare flexible objects to facilitate their tasks, such as stretching a shirt before folding it, or bending a string during knitting to pick it up with the needle [53,54].

The temporal evolution of the cup and ball is sensitive to initial conditions and chaotic behavior is possible as it is a nonlinear dynamical system [8,19,20,55,56]. Chaotic behavior is indicated in the stroboscopic plot of Fig 8 that displays an intricate pattern of interaction forces resembling the period-doubling bifurcations of one-dimensional nonlinear maps (see also Additional Analysis) [48,49]. Quasi-periodic and chaotic behavior can be unpredictable, even in a purely deterministic system, and for the human performer it is impossible to predict. Applying real-time corrections based on perceived errors is problematic due to delays in the feedback loop that have longer timescales than the rapidly changing behavior in the hand and object interactions [27,57,58]. Hence, we argue that subjects learn to initialize the ball states to increase predictability of the interactive dynamics. Appropriate initialization of an object to achieve more ‘comfortable’ interaction with it is also reminiscent of the “end-state-comfort” effect; humans tend to grasp objects with hand configurations that facilitate more comfortable final postures at the end of the object transport [59,60]. While a number of studies reported this finding, comfort and personal preference is a subjective concept. The present study used a quantitative approach based on interactive dynamics to show that preparation—setting initial conditions—is important to achieve a task goal successfully.

#### Preparation versus exploration

Results clearly showed that all subjects converged to initial ball states. However, individual subjects chose different initial ball angles indicating that the initial ball angle was not determined by the dynamics of the object, but that there was a choice. As the simulated solution space for transient duration showed, there was a manifold of ball states that could achieve transients with zero duration. The final values of ball angle ended up in this manifold (Fig 9A). Hence, all subjects prepared themselves for the task in different but successful ways.

Analysis of the preparation interval exhibited a significant decrease, paralleling the convergence to the appropriate initial ball states. In addition, the cup cycles during the preparation interval, where no instructed frequency was present, revealed a decreasing range of cycle frequencies. In later trials subjects relatively quickly created cup movements of the desired 0.60 Hz frequency, i.e. prepared themselves for the upcoming task. After approximately half of the trials, subjects adopted the task-instructed cup frequency and initial conditions in a relatively short time. These findings reflect that initially subjects explored the system, i.e., performed system identification, to find the best preparation. Hence, they learned to prepare the object without the need for further exploration. While we did not further scrutinize the relative contribution of exploration versus preparation, this distinction between exploration and preparation is important, given the wide-spread interest in exploratory movements. Exploratory movements are generally regarded as random aiming to identify optimal locations in solution space [29,61]. In contrast, preparation is goal-oriented, assuming the existence of favorable conditions to execute a task optimally.

#### Initializing actions in neural systems

While it is a challenge to relate our behavioral findings to their neural substrate, recent studies in cortical dynamics of non-human primates have provided evidence that neural populations show distinct preparatory behavior [62,63]. Simultaneous electrophysiological recordings in motor and pre-motor areas showed that prior to movement onset, spontaneous firing transitioned into a movement-specific preparatory state with stable firing rates [64–66]. However, these observations of neural initial conditions were observed during a delayed start of a reaching movement, i.e., in a time interval where no movement had yet occurred. It is nevertheless intriguing to speculate that cortical networks in the brain may converge to initial conditions in the context of preparing for a complex action such as transporting an object. Such preparatory strategies may be employed at different levels, e.g. neural and behavioral.

### Transients, transitions, and stability

The duration of start-up transients clearly decreased across trials in all subjects as hypothesized. Even though the calculation of transient duration from the data relied on tuning a threshold, the decrease in duration was clear and this decline did not change much with different thresholds. This highlights the significance of initializing the object dynamics to reach a predictable steady state as soon as possible.

Transient is a concept in nonlinear dynamics that describes the time the system takes to arrive at an attractor. The duration is dependent on the so-called basin of attraction, both on its boundaries and the steepness of the gradient [32,67]. As such, transients may indicate the strength or degree of stability of an attractor. Very few studies to date have paid attention to transient behaviors, as the typical focus in movement control is either on steady state behavior or on learning curves across trials. Non-stationary behavior is hard to quantify as it does not permit meaningful averaging. However, transient dynamics when starting from rest can be revealing. For example, when tracking a periodic signal with a cursor, humans tracked the signal with a constant phase shift that shortened the transient when the target signal changed its period [68]. The same behavior was reported when tapping in synchrony with a metronome [69].

Transitions between two stable states are different from start-up transients, but similarly informative about control and coordination. Sternad and colleagues examined involuntary transitions between discrete point-to-point arm movements to smooth rhythmic behavior when paced by a slowly accelerating or decelerating metronome frequency [70,71]. It was the changing kinematics, not the steady state behavior, that provided insights into principles of coordination. Transitions between different modes of oscillations in a rhythmic movement (non-equilibium phase transitions of a nonlinear dynamical system) were central in a seminal study by Kelso and colleagues [72]. When moving left and right fingers rhythmically in anti-phase mode, increasing frequency induced a transition to in-phase mode [73]. This spontaneous transition and the frequency at which this change occurred provided important information for characterizing the system’s dynamics [74]. When subjects intentionally switched from anti-phase to in-phase behavior [75,76], ‘switching time’, or the duration of the transient, served as a measure to assess the differential stability of the two attractors. In sum, non-stationary behavior can provide an interesting window into control and coordination.

### Increasing predictability via stability

The present findings on the steady state provided support for the overarching hypothesis that humans create predictable interactions, reinforcing our previous work on the same cup-and-ball task that increasing predictability in interactions is an implicit task goal [19,22,77]. In order to further specify the strategies that ensure predictability, Bazzi and Sternad previously demonstrated that dynamic stability measured by contraction [78] is a means to achieve predictability [26]. When a dynamic system is at a stable attractor, small perturbations return to the limit set without requiring explicit corrective control [79]. Stability thereby obviates excessive error corrections as previously argued in the context of the task of rhythmically bouncing a ball [80–82]. Using a discrete cup-and-ball movement, where subjects learned to accommodate a visible perturbation that could cause the ball to escape, Bazzi et al. showed that subjects learned to approach the visible ‘bump’ with a strategy that exploited the contracting regions of the state space [26,83]. Given this robustness to perturbations, it is reasonable to conclude that a stable state requires decreased cognitive or computational effort.

Stability is usually measured by applying perturbations and assessing the time to return or ‘relax’. Alternatively, variability can also express the degree of stability of a dynamical system. In the literature on bimanual rhythmic movements, relative phase between two rhythmic components served as the variable that characterized the stability of two coupled oscillators. Mean relative phase and its variability quantified the degree of stability of the coupled system. Based on this research, we used relative phase between the two oscillatory cup and ball movements to indicate when transients ended and stable steady state began. Taking the difference between the two phases of the two components subtracts out large amplitude fluctuations, allowing the signal to reveal small fluctuations. The same signal also revealed the changing stability of the steady state. As hypothesized, the fluctuations around the stationary signal decreased with practice.

### Predictability, minimum intervention principle, and uncertainty

The reason for seeking predictability is that it simplifies the challenge of prediction: it obviates the need for precise internal models. More economical predictions may lead to fewer errors, hence afford reliance on feedforward control without excessive feedback-based error correction. Although effort minimization and the minimum intervention principle are core to stochastic optimal feedback control models [44,84], they are quite distinct from predictability. The former ensures that deviations from typical behavior are only corrected if they interfere with the goal of the task, hence, leading to minimal effort expenditure and synergetic coordination. However, the hypothesis of predictability is not concerned with how error corrections are selected to channel variability *during* the movement execution, rather, it examines how subjects *prepare* the initial conditions of an object *prior to* the intended movement to ensure simpler interactions with the object during the movement. Once a strategy is learned based on the objective of increasing predictability, one could argue that the variability around such strategy *during* the movement could be dealt with based on the minimum intervention principle. However, this was not tested in our study.

Note that increasing predictability is also similar, but not equivalent to, reducing uncertainty. The latter implies a reduction in noise in a stochastic system, whereas predictability is also a feature of deterministic dynamics. A deterministic system can exhibit chaos that can manifest as variability in its time series and easily be mistaken for stochastic noise [85]. Our model simulations are purely deterministic and can exhibit fluctuations of different degrees; however this is not random noise, i.e., uncertainty, but has a complex structure (as revealed in Fig 8). The human data likely present a combination of deterministic structure and random noise.

### Inverse and forward simulations and their model assumptions

As a complementary avenue to evaluate whether subjects’ initial explorations and preparations had significant effects on simplifying dynamics or other objectives, inverse and forward dynamics simulations were performed. To start, the inverse dynamics simulations made no assumptions about the human controller; they only determined the force input needed to achieve sinusoidal cup trajectories. The equations to solve for *F*_*inter*_(*t*) did not take into account the hand on the cup, which introduced impedance and therefore increased the order of the system. Nevertheless, the simulation results visualized how the potential complexity of the interaction forces depended on the initial ball states. Mutual information between these interaction forces and the object dynamics rendered matching results to express the degree of predictability for given initial ball states. Subjects’ preferred initial ball states at the end of practice approximately aligned with the values that achieved more predictable interactions in simulation. This coincidence is noteworthy for yet another reason: the inverse dynamics simulations did not include any online error corrections based on feedback. Therefore, this suggests that subjects could accomplish the task without significant online error corrections. To reiterate, online error corrections suffer from long delays in the sensorimotor system and rely on prediction of sensory consequences. It may be difficult to predict sensory consequences for unpredictable interaction forces.

The forward simulations served to establish a more direct link between initial conditions and the hypothesized control objectives: reduced transient duration and steady state stability, but also minimal effort, jerkiness, and risk. As a controller is needed, we chose a relatively simple impedance controller, following a sinusoidal desired trajectory, that served to minimize deviations from the desired sinusoidal cup trajectory. The impedance is a proxy for mechanical or spinal reflex mechanisms with short latencies between 25 to 80 ms [86,87]. In contrast to explicit error corrections in a biological system that involves transcortical sensorimotor loop delays >100 ms, impedance-based corrections are regarded as compensations around a planned sinusoidal zero-force trajectory that are either spinally mediated or that arise from intrinsic muscle mechanics [88,89]. While the constant impedance values assumed in this task are likely to be an oversimplification of the biological system, reliable estimates from real human movements are still missing [90,91]. Variations of the impedance values and also additional feedback terms with delays and noise could be introduced. However, the model predictions with this simple model accounted remarkably well for the experimental results.

The solution spaces generated by the impedance controller for all hypothesized objectives reinforced the experimental findings. The broad distribution of initial ball angles in the data matched the redundancy in the solution space for the transient durations, showing a manifold of transients with zero duration. This may explain why individual subjects did not converge to the same initial conditions. While they had a choice, the initial states were largely within this manifold. The stability estimates indeed followed the gradient of the solution space approaching optimal values of stability. In contrast, the solution spaces for effort, smoothness and risk showed little congruence with the data. It should be pointed out that the parameters for the impedance controller were identical for all simulations. Some of these results appear counter-intuitive. Effort indeed increased, consistent with previous demonstrations [19,22]. The decrease in smoothness of the cup kinematics can be understood from detailed inspection of the cup’s trajectories: at each excursion, the ball introduced an additional extremum, appearing as an additional frequency in the profile. This increased the ‘peakiness’ of the spectrum leading to a less smooth SPARC metric. The risk metric proved less informative. Further analyses showed that risk was highly correlated with cup frequency. As cup frequency showed a small increase throughout the experiment, risk increased almost proportionally.

### Interrelation between objectives?

Could the different objectives be interrelated? For example, the solutions for short transients and optimal stability partially overlap. Might subjects trade one objective for another? To begin, the six objectives are indeed not independent as they are all mathematically derived from simulating the controlled system based on initial conditions: ball position and ball velocity. However, the topologies of the resulting landscapes are very different, which would make combinations and trade-offs relatively complex. A trade-off is also unlikely as the three ‘predictability’ metrics, mutual information, transient duration and variability of relative phase all show an improvement, while all three alternative objectives get worse. Nevertheless, one might consider that there may be one cost function that combines all six objectives, essentially a weighted sum of the objectives, that afford one global minimum. However, quantitative evaluation of such a combined solution space would be difficult as the six costs would require normalization and weighting to create a single cost function, inviting arbitrariness in determining the weights.

### Sensory information or reinforcement learning to achieve predictable dynamics?

So far, our study has only documented that predictability is an important objective in complex dynamic interactions and that appropriate initial conditions shorten transients to allow a more stable and predictable steady state to emerge earlier. However, we have not yet addressed, let alone answered, the question of *how* humans sense and attain these predictable interactions. It is unquestioned that visual, haptic and proprioceptive feedback may play an important role in any interaction with an object in the environment, and studies of their relative contributions to control and learning abound [81,92–95]. Undoubtedly, subjects used visual, haptic, and auditory information in the present experiment to improve from one trial to the next. Even though no explicit performance score was provided, subjects could hear whether they were in synchrony with the metronome; they could see the ball motion in response to their interaction forces, and they could feel the ball forces against their hand. The system’s behavior is probably learned by exploring the object’s response to different human force inputs, likely in a trial-and-error fashion using reinforcement learning. Feedback from each trial may have then been used to improve performance on the next trial, maybe even from one cycle to the next.

But did subjects use sensory information for online control and correction? Assuming 200 ms as the minimal sensorimotor loop time, any error correction based on sensed events 200 ms in the past may not sufficiently compensate for the rapidly evolving dynamics [96]. Hence, for any feedback-based error corrections, the system’s states have to be predicted and control needs to use predicted motor and sensory consequences, as recognized previously [97–101]. Note that the inverse dynamics simulations were done without any error corrections and reasonably predicted the experimental initial ball states, indicating that subjects could have accomplished the task largely without online error corrections. As shown, objects with nonlinear internal dynamics can exhibit chaotic dynamics that is essentially unpredictable [19,102]. Hence, the complex evolution of the hand-object interactions may be too challenging to accurately predict over a window of 200 ms. Control of systems with time delays from multiple sources in a distributed system becomes a problem of infinite dimensionality [103,104]. How do humans control such infinite-dimensional unpredictable objects? How do they represent the complex interactive dynamics? Our study only showed that humans simplified the interactive dynamics by preparing the object with appropriate initial conditions and making interaction forces predictable. The question how humans achieve predictability is still unanswered and needs further investigation.

## Conclusions

This study investigated rhythmic physical interaction with a dynamically complex object. Results indicated that when given the opportunity to choose and exploit the initial conditions of the system, subjects did so in a manner that simplified interaction dynamics, characterized by transient durations and predictability of hand-object interactions. The initial conditions that were predicted by model simulations to decrease transients and increase mutual information between hand and object were consistent with those chosen by subjects. These results support the hypothesis that predictability of an object’s dynamics is a principal objective of human control strategies. Predictable interaction forces may be less computationally taxing on sensorimotor information processing.

Manual dexterity in hand-object interactions–tool use–is essential in activities of daily living and impairments have detrimental effects on the quality of life. Our findings can shed light on the coordinative challenges and set the stage for a better understanding of impaired dexterity. Moving a cup filled with coffee is an ecologically valid control challenge, inherent in many self-feeding activities. The experimental platform and theory-based analyses and insights create new potential for assessment and therapy for clinical populations. To date, clinical assessments have been confined to kinematics and simple descriptive outcome measures, like error scores or completion times. More precise description of functional manual interactions requires a validated theory. This research is a step in that direction.

## Methods

### Ethics statement

Prior to the experiment, all subjects gave written informed consent and verified that they understood the written and oral task instructions. All procedures were approved by the Northeastern University Institutional Review Board (IRB#:10-06-19).

### Participants

Thirteen college-aged adults (8 male, 5 female) with no self-reported neuromuscular pathology volunteered for the experiment. All participants self-reported to be right-handed and subsequently performed the task with their right hand. All were naive to the experimental task and the purpose of the study.

### Experimental task and model

The task of ‘carrying a cup of coffee’ was simplified to that of pushing a two-dimensional arc with a ball sliding inside along a horizontal line (Fig 1A and 1B). Since the ball was sliding instead of rolling, the system was mechanically equivalent to a cart sliding on a frictionless line with a suspended frictionless pendulum. The pendulum bob was represented by the ball, and the cup position corresponded to the cart position. In addition, the arc of the cup aligned with the rotational path of the pendulum. The equations of motion for this relatively well-known “cart-and-pendulum” model are:

$$[m_{c}+m_{p}]\ddot{x}=m_{p}l[\dot{\theta^{2}}\;\sin\;\theta -\ddot{\theta }\;\cos\;\theta ]+F_{inter}=F_{ball}+F_{inter}$$ (2)

$$\ddot{\theta }=-\frac{\ddot{x}}{l}\;\cos\;\theta -\frac{g}{l}\;\sin\;\theta$$ (3)

where *x* was the cup position and *θ* was the ball angle. The downward vertical orientation of the ball defined zero angle, with counterclockwise rotations denoted as positive. The mass of the cup was denoted by *m*_*c*_ and the mass of the ball by *m*_*p*_. *F*_*inter*_ was the interaction force applied by the subject’s hand acting on the cup. *F*_*ball*_ was the force applied by the ball onto the cup, which was transmitted as haptic feedback to the hand of the participant via the robot’s handle (Fig 1C). The parameter values of the cup-ball-system were: *m*_*c*_ = 2.40 kg, *m*_*p*_ = 0.60 kg, pendulum length was *l* = 0.45 m, and gravitational acceleration *g* = 9.81 m/s^2^. The values were selected to be large enough that subjects felt the forces generated by the ball, while also small enough that the subjects did not fatigue. Despite these simplifications introduced by the model, the challenges of “carrying a cup of coffee” were retained: underactuation and nonlinearity.

### Virtual rendering with visual-haptic interface

The cup-and-ball system was implemented as a virtual environment. For the rendering, the cup and ball were displayed on a large projection screen (Fig 1C and 1D). Subjects controlled the horizontal cup displacement via moving the handle of an admittance-controlled robotic manipulandum (HapticMaster, Motekforce, Netherlands). The force applied by the participant on the manipulandum *F*_*inter*_ was converted into displacement *x* of the virtual cup [105]. Using the cup-and-ball model, a custom-written C++ program based on the HapticAPI computed the ball kinematics that then controlled the ball in the virtual display and fed the ball force back to the subject’s hand in real-time with 120 Hz processing speed. Since the cup was limited to move along a horizontal line, the movements of the robotic arm were restricted to horizontal linear translations parallel to the participants’ frontal plane. The applied force *F*_*inter*_, the cup position *x*, velocity $\dot{x}$, and acceleration $\ddot{x}$, and the computed angular position *θ*, velocity $\dot{\theta }$, and acceleration $\ddot{\theta }$ of the ball were recorded at 120 Hz.

### Visual display

The visual display showed the cart as a yellow arc that represented the cup and a small white circle that represented the ball; two blue rectangular targets at each end of the horizontal line, Box A and Box B, delimited the displacement of the cup (Fig 1D). The visual displacement of the cup on the screen was 4 times larger than the physical displacement of the robot. The physical distance between the centers of Box A and Box B was 0.30 m and their centers were located at -0.15 m and 0.15 m, respectively, with zero defined half-way between them. Since the pendulum length *l* of 0.45 m would have generated a very large cup, the visualization of the cup on the screen was 7.5 times smaller than the radius of the simulated cup. The cup’s rim was at ±50 deg such that whenever the ball angle exceeded this angle, the ball would ‘spill’ and visually fall out of the cup, resulting in a failed trial.

### Experimental procedures

Participants sat in a chair approximately 2 m in front of a projection screen and grasped the handle of the robotic manipulandum (Fig 1C). They were seated with their sternum aligned with the robot; the robot’s base was equidistant between Box A and Box B on the screen and the HapticMaster arm was orthogonal to the screen. At the beginning of each trial, the cup was positioned in Box A with the ball resting at the bottom of the cup (*θ* = 0 deg). Subjects were instructed to move the cup in a regular rhythm between the two target boxes following a metronome. Its frequency was set to 0.60 Hz which was close to the anti-resonance of the cup-and-ball system at 0.74 Hz. Theoretically, cup movements at this frequency are canceled by the ball motion. Any movements close to this frequency become difficult as chaotic behavior is elicited. In a previous study where subjects could select their preferred frequency, they clearly avoided the anti-resonance and their variability increased closer to this frequency [40].

Prior to starting the rhythmic movements subjects were asked to explore the cup-and-ball dynamics by ‘jiggling’ the cup back and forth; they could take as much time as they wanted. Subjects were told to ‘jiggle’ in such a manner that best prepared them for the following rhythmic movements at the given metronome pace. The instruction specified that these preparatory movements should be confined to the left half of the total cup amplitude. No further instructions were given about how to entrain with the metronome.

Once subjects felt ready, they moved the cup towards Box B and continued moving in rhythmic fashion between the two boxes without losing the ball (|θ|<50 deg). The metronome only started when the participant reached Box B for the first time to ensure that subjects could entrain to the metronome from the beginning without further adjustments. After that the metronome continued at 0.60 Hz to pace their movements for 15 s. At the end of the trial a success sound was played if the trial was completed without losing the ball. The robotic manipulandum stiffened so that the subject could not continue moving the cup. If the ball was lost, it visually fell out of the cup and a failure sound was played and the trial ended shortly thereafter. Subjects used the continuous visual feedback from the screen, haptic feedback from the robotic manipulandum, and auditory feedback from the metronome beeps to form their judgement about their performance. The entire experiment session was comprised of 4 sets of 30 trials each and lasted approximately one hour. Subjects were given breaks of at least 5 minutes between each set to avoid fatigue.

### Analysis of experimental data

To provide a general description of subjects’ performance, the data were scouted for failed trials, i.e., when the ball angle exceeded the rim angle and escaped from the cup. Two of the 13 subjects lost the ball in 55 and 60, or 46% and 50% of their trials, respectively. These numbers were higher than 1.5 times the third quartile of all 13 subjects’ failure rates; hence, the two subjects were excluded as outliers from all further analysis [41]. For the remaining 11 subjects, movement frequency and amplitude were calculated to test whether or how well subjects satisfied the instructions. For these calculations their failed trials were excluded. Note that due to these failed trials, especially in the beginning, these means and standard errors across subjects often comprised fewer than 11 data points. In addition, the pairwise comparison of early versus late performance was computed using the first 5 and last 5 successful trials of each subject (that may have spanned across more than 5 trial numbers).

#### Preparation interval and initial conditions

Exemplary raw time series of cup position, cup velocity, ball angle and ball velocity for 60 trials of one subject are shown in Fig 2A. For better visibility, every other trial was omitted; the color gradient indicates the sequence of trials; blue marks early trials, green later trials. The time series were aligned at time *t* = 0 defined as the time when the cup velocity was zero before the subject initiated the first full-amplitude movement to Box B. The interval from the beginning of the subjects’ movements to *t* = 0 defined the preparation interval. The variables that were used to test the hypotheses were initial conditions, transient duration, and mutual information of different trial segments.

In the beginning subjects moved the cup back and forth with varying amplitudes and frequencies to explore the ball dynamics. The last point of zero cup velocity before the cup started the first full amplitude to Box B was the time point at which the initial conditions were defined (Fig 2A). At *t* = 0, initial ball angle, ball velocity, and cup position were taken; cup velocity was zero by definition.

#### Mutual information

Mutual information (*MI*) was calculated over the entire trial, starting at *t* = 0 and ending after 15 s. *MI* is an information-theoretic measure that quantifies the degree of information that can be attained about one variable by observing another variable. Therefore, *MI* measures predictability of a signal [106,107]. High *MI* indicates that a high degree of information is known about one random variable given the knowledge of another random variable. Unlike cross-correlation, *MI* can evaluate both linear and nonlinear dependencies [108,109]. In our data, *MI* quantifies the predictability between the input, i.e., interaction force *F*_*inter*_, and the output of the system, cup trajectory and ball trajectory. *MI* measures to what degree the dynamic behavior of the system diverged from simple periodic input-output mapping [19]. Since the cup and ball trajectories were close to sinusoidal, the kinematics could be represented by their phase in space:

$$\mathrm{cup}\;\mathrm{phase},\;\varphi_{cup}(t)=\arctan[\frac{\dot{X}}{\left(2\;\pi \;f\;X\right)}],\;and\;ball\;phase,\;\varphi_{ball}(t)=\arctan\left[\frac{\dot{\theta }}{\left(2\;\pi \;f\;\theta \right)}\right].$$

Then, mutual information was obtained by the following calculation using

$$MI(\varphi ,F_{inter})=\iint \;P\;(\varphi ,F_{inter})ln\left[\frac{P(\varphi ,F_{inter})}{P(\varphi )P(F_{inter})}\right]\;d\varphi \;dF_{inter}$$ (4)

where *P* denotes the probability density functions for *φ*(*t*) and *F*_*inter*_(*t*). Mutual information is a dimensionless quantity, and its unit depends on the base of the logarithm that is used. Here, the natural logarithm was used, and the unit of mutual information is the nat.

#### Transient duration and steady state: Relative phase

As the movement started, it underwent a transient before settling into a steady state. To calculate the duration of the transient, a steady state for the cup-and-ball movements had to be defined first. For sinusoidal cup movements at frequencies less than the pendulum’s natural frequency at 0.74 Hz, the cup and ball were in-phase, i.e., had zero relative phase (Fig 2B). Hence, to identify where the transient ended and the steady state began, the instantaneous phase for the cup and, separately, for the ball position was computed using the Hilbert transform [110,111]. The difference between the two phases yielded the continuous signal of relative phase.

To obtain instantaneous phase of the cup and ball trajectories, the Hilbert transform was computed for the entire trial, except the last 1 s to eliminate artifacts due to edge effects. As any periodic function can be expressed in the complex plane, the measured displacement signal *S*(*t*) can be written as: ζ(*t*) = *S*(*t*) + *iS*_*H*_(*t*), where *S*_*H*_ (*t*) is the Hilbert transform of *S*(*t*), and *i* is the imaginary number. By Euler’s formula, the periodic function in the complex plane is rewritten in terms of amplitude *A*(*t*) and phase *φ*(*t*): ζ(*t*) = *A*(*t*)*e*^*i*φ(*t*)^. This equation shows that the signal consists of two separable functions of time, *A*(*t*) and *φ*(*t*). The instantaneous phase *φ*(*t*) can be represented in continuous or modular form between 0 and 2π (Fig 2B). These calculations were performed in MATLAB using the function hilbert.m (The Mathworks, v.29b, Natick MA). To obtain relative phase, the algebraic difference between cup phase and ball phase was computed. The calculated relative phase was subsequently filtered using a 5 Hz lowpass 8th-order Butterworth filter.

The bottom panel of Fig 2B shows an example time series of relative phase and how it settled into an approximately constant value of zero. To define the end of the transient and the beginning of the steady state, a threshold had to be determined. Various thresholds were tested, and the best value was 15% of its maximum value of 180 deg, i.e., 27 deg. Thresholds more stringent than ±27 deg often led to transients of 15 s, i.e., the entire trial. The time point when relative phase was smaller than 27 deg and remained below this threshold for the rest of the trial defined the start of the steady state.

#### Variability of relative phase as a measure for stability

To obtain an estimate of the stability of the steady state portion of the trial, the standard deviation of relative phase SD *ϕ* has been used in previous studies on coupled oscillations [43,112,113]. It was calculated as follows:

$$SD\phi =\sqrt{\frac{1}{N-1}\sum_{i=1}^{N}{\left|\phi_{i}-\mu \right|}^{2}}$$ (5)

where *μ* is the mean of *ϕ*, the relative phase during the steady state interval; *N* is the number of samples of *ϕ* during the steady state interval.

#### Root mean square of force as a measure for effort

The force of the hand acting on the robotic manipulandum, *F*_*inter*_ was measured using a force sensor at the base of the robot handle. *F*_*inter*_ was used to quantify effort exerted during each trial, calculated as the averaged integrated squared force, root mean squared force, which quantified *Effort*.


$$Effort=\frac{1}{T}\int_{0}^{T}{\left[F_{inter}(t)\right]}^{2}\;dt$$ (6)

*T* is the duration of the trial, 15 s.

#### Smoothness of cup and ball trajectories

Previous work on human control of a mass-spring system found that humans prioritize smoothness of the object kinematics [13]. This prior study assessed smoothness based on minimum crackle, two derivatives higher than jerk, to evaluate their point-to-point transport movement. Their calculation was constrained by the boundary conditions of the discrete movement, specifically the hand had to be at rest (zero acceleration) at the start and end of the reach. Such boundary conditions are not present in a continuous oscillatory interaction, making this metric unsuitable for continuous rhythmic movements. The Spectral Arc Length (SPARC) method, developed by Balasubramanian et al. [46,47] rendered more consistent results for continuous rhythmic movement and was also less affected by noise.

The SPARC method calculates smoothness in the frequency domain, specifically measuring the arc length of the normalized Fourier magnitude spectrum of the speed profile [46]. A movement that is smooth in the time domain shows a limited number of frequency components in the Fourier domain with a less complex envelope with a shorter arc length. Thus, for a speed profile *v*(*t*), *t*∈[0,*T*] with movement duration *T*, the arc length of the frequency-normalized Fourier magnitude spectrum is calculated as:

$$\eta_{sparc}≜-\int_{0}^{\omega_{c}}{\left[\sqrt{{\left(\frac{1}{\omega_{c}}\right)}^{2}+{\left(\frac{d\hat{V}(\omega )}{d\omega }\right)}^{2}}\right]}^{\frac{1}{2}}d\omega ;\;\hat{V}(\omega )≜\frac{V(\omega )}{V(0)}$$ (7)

$$\omega_{c}≜\{\omega_{c}^{max},min\{\omega ,\hat{V}(r)<\overset{-}{V}\forall r>\omega \}\}$$ (8)

*V*(*ω*) is the Fourier magnitude spectrum of *v*(*t*); $\hat{V}(\omega )$ is the amplitude spectrum, normalized with respect to the value at 0 Hz (the DC component). The first term under the square root normalizes the arc length with respect to *ω*_*c*_, the final frequency that the spectrum is calculated over. *ω*_*c*_ is adaptively selected based on a threshold $\bar{V}$ and is upper-bounded by $\omega_{c}^{max}$. The adaptive frequency window allows the smoothness metric to be independent of movement amplitude and duration. Following [46], these two parameters were set to $\bar{V}=0.05$ and $\omega_{c}^{max}$ = 20*π*. A higher value of $\bar{V}$ would result in a lower cut-off frequency and eliminate details in the spectrum beyond the cut-off; a lower value of $\bar{V}$ would make the metric sensitive to noise.

As movement reversals can be interpreted as intermittencies in the movement, smoothness of rhythmic movement must be calculated over individual movement cycles, delimited by minima of the cup position. The average smoothness for the cup or ball kinematics for each trial Λ was calculated as:

$$\Lambda =\frac{\sum_{i=1}^{N}\frac{\lambda_{i}}{d_{i}}}{N}$$ (9)

*λ*_*i*_ is the smoothness measure of one cycle, normalized by its duration *d*_*i*_; *N* is the total number of cycles per trial.

#### Energy margin as a measure for risk

A core feature in this experiment is that the ball can escape if the ball angle exceeds the cup’s rim height. For the ball to not spill from the cup, the total energy of the ball should not exceed a threshold energy or escape energy. The system’s escape energy is the maximum possible energy of the ball before it escapes the cup. The escape energy is determined by the parameters of the system: cup rim angle, pendulum length, ball mass and gravity. If the total energy of the ball exceeds the escape energy, the ball will fly out of the cup, unless the energy is dissipated. The energy margin is the difference between the escape energy and the total energy of the system at any given time:

$$E_{Kinetic}=\frac{{\dot{\theta }(t)}^{2}l^{2}m}{2}$$ (10)

$$E_{Potential}=mgl(1-\cos\left(\theta (t)\right))$$ (11)

$$E_{Total}=E_{Kinetic}(t)+E_{Potential}(t)$$ (12)

$$E_{Escape}=mgl(1-cos\theta_{Escape})$$ (13)

$$E_{Margin}(t)=E_{Escape}-E_{Total}(t)$$ (14)

This yields a continuous timeseries of *E*_*Margin*_(*t*) over the trial. Risk was evaluated at the moments of ball angle extrema, i.e., the moments where the ball was close to escape. These point estimates were summed over the trial duration. Risk was calculated as 1 minus the energy margin:

$$Risk=1-\left(\frac{1}{T}\right)\left(\frac{1}{E_{Escape}}\right)\int_{0}^{T}E_{Margin}(t)\;dt$$ (15)

### Model simulations

#### Inverse dynamics calculations

Inverse dynamics calculations were conducted to test the effect of the initial conditions on the interaction force required to generate the desired rhythmic movement of the cup and ball. These calculations did not assume any controller but computed the temporal profile of *F*_*inter*_(*t*) needed to generate a sinusoidal cup displacement with peak-to-peak amplitude *A* and frequency $f:\;x(t)=\left(\frac{A}{2}\right)\;\sin\;\left(2\pi ft+\frac{\pi }{2}\right)$, where *t* was time. As initial ball angle appeared to show the most pronounced convergence to specific values, the inverse dynamics calculations were run with different initial ball angles *θ*_0_, all other initial conditions fixed. A second inverse dynamics simulations fixed *θ*_0_, but varied ball velocity $\dot{\theta_{0}}$. Results are summarized in the Additional Analysis.

For the simulations the initial condition for the cup position *x*_0_ was set to -0.15 m, the left-most position for the cup, and initial cart velocity $\dot{x_{0}}$ was set to 0. Initial ball velocity $\dot{\theta_{0}}$ was set to -15.81 deg/s, the average initial ball velocity across subjects. While these initial values were kept constant, the calculations were conducted for all *θ*_0_ between -90 deg and 90 deg; the step size was 5 deg. To best compare the simulations to subject data, the sinusoidal cup frequency *f* was set to the subjects’ mean frequency 0.58 Hz, and the peak-to-peak amplitude *A* was set to the subjects’ mean amplitude 0.308 m. Fig 8A and 8B show two example time series of *F*_*inter*_(*t*) and ball angle *θ*, obtained for a sinusoidal cup position *x*, simulated with two different initial ball angles. To summarize how the temporal profiles of *F*_*inter*_(t) depended on *θ*_0_, the time series of *F*_*inter*_(t) were strobed at the maxima of the simulated cup position trajectory. The marginal distribution of these strobed values reflected the regularity of the interaction force. To summarize the results for all initial ball angles, the marginal distributions were plotted as a function of *θ*_0_. To express the degree of complexity of each distribution, mutual information was calculated between the computed force profile and the ball dynamics, following the same procedures as for the experimental data.

In a second inverse dynamics simulation, initial ball velocity values $\dot{\theta_{0}}$ were varied between -180 deg/s and 180 deg/s; the step size was 5 deg/s. The initial cup position *x*_0_ and velocity $\dot{x_{0}}$ were identical to above; *θ*_0_ was one of four values *θ*_0_ = 15, 0, -15, -30.29 deg (the simulations were run four times). For each simulation run with a given $\dot{\theta_{0}}$, *F*_*inter*_(*t*) was strobed at the maxima of the simulated cup trajectory and its marginal distribution was obtained; these distributions were plotted against initial ball velocity, as shown in the Additional Analysis.

#### Forward dynamics simulations with impedance control

**Fig 11:**
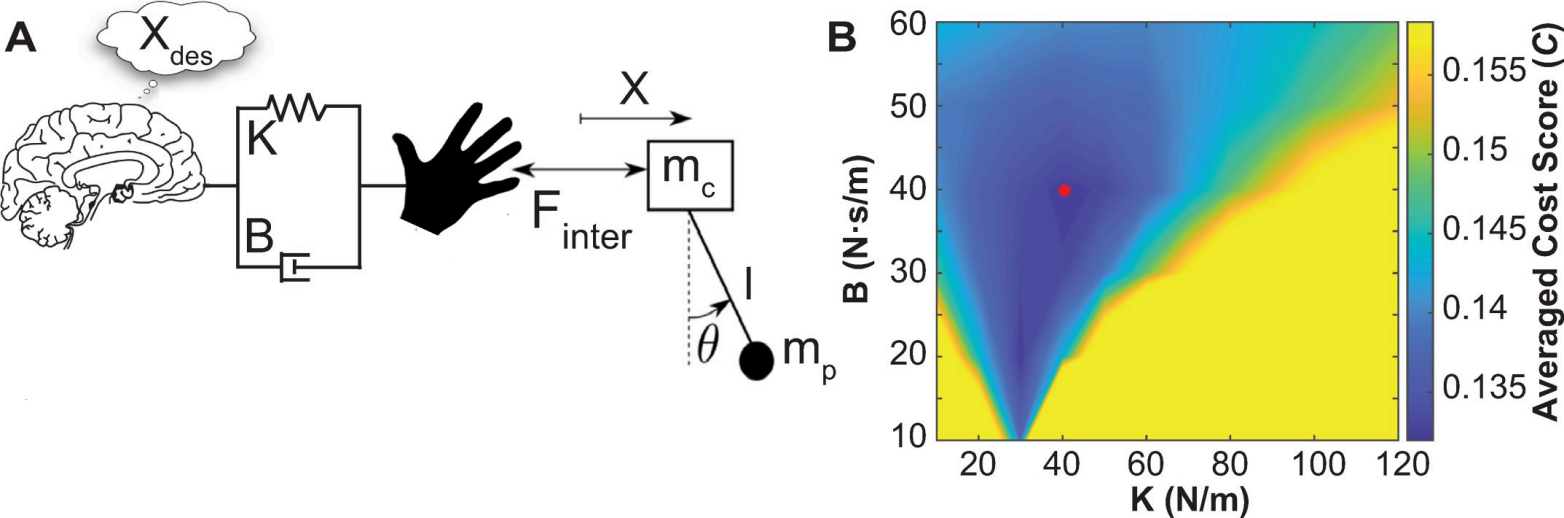
**Model of hand impedance and estimation of impedance values: A.** Control model with a first-order hand impedance coupled to the cup-and-ball system. The interactive dynamics is represented by a spring *K*, in parallel with a damper *B*. The desired trajectory is denoted as *x_des_*. **B.** Landscape of cost scores *C* for all stiffness and damping combinations, fitted to the experimental data. *C* is the goodness of fit for given pairs of *K* and *B* to the human data. Yellow indicates impedance values that produce high *C* values and blue values that produce low *C* values. The global minimum at stiffness *K* = 40 N/m and damping *B* = 40 Ns/m, shown by the red point. This landscape is the point-wise average of all 1100 successful trials.

To evaluate and compare the chosen initial conditions with a control model, forward dynamic simulations were conducted. However, this required a controller. To minimize the number of parameters, a first-order impedance controller with a spring and damper in parallel was used as force generator *F*_*inter*_ around the desired cup position *x*_*des*_ and velocity ${\dot{x}}_{des}$ (Fig 11A):

$$F_{inter}=-K(x-x_{des})-B(\dot{x}-{\dot{x}}_{des})$$ (16)

Stiffness *K* and damping *B* served to minimize errors between *x*(*t*) and *x*_*des*_(*t*) and $\dot{x}(t)$ and ${\dot{x}}_{des}(t)$, respectively. The desired trajectory was a sinusoid, sufficient to represent the instruction to move the cup rhythmically. The parameters *K* and *B* were assumed to be constant during a trial. However, appropriate values that matched the experimental data had to be specified.

#### Estimation of human stiffness and damping values

Given the yet unresolved problem of directly measuring stiffness and damping during movement, *K* and *B* were estimated post hoc from the subjects’ kinematic data. Using an optimization procedure, the position and velocity of cup and ball of each experimental trial were compared to the corresponding trajectories generated by feedforward simulations, sweeping through the 2-dimensional *K-B* parameter space [22]. For each experimental trial, the mean frequency *f* and mean amplitude *A* were calculated, and the simulation was performed with the same *f* and *A* of the given trial. The simulations also started with the same initial conditions as the experimental trial, using *θ*_0_ and $\dot{\theta_{0}}$; *x*_0_ was set to -0.15 m, ${\dot{x}}_{0}$ was zero by definition. The simulations were run for 15 s with different combinations of *K* and *B* sweeping through the following ranges *K*: 10 N/m to 120 N/m, step size 2 N/m; *B*: 10 Ns/m to 60 Ns/m, step size 1 Ns/m. The cost *C* to be minimized was the root mean square difference between all simulated and experimental trajectories *x*(*t*), $\dot{x}(t),\theta (t),\dot{\theta }(t)$. Each term was normalized by the corresponding experimental maximum value (H_∞_ norm):

$$C=\frac{1}{4}\left[\frac{rms(x_{e}-x_{s})}{{\left\|x_{e}\right\|}_{\infty }}+\frac{rms({\dot{x}}_{e}-{\dot{x}}_{s})}{{\left\|{\dot{x}}_{e}\right\|}_{\infty }}+\frac{rms(\theta_{e}-\theta_{s})}{{\left\|\theta_{e}\right\|}_{\infty }}+\frac{rms({\dot{\theta }}_{e}-{\dot{\theta }}_{s})}{{\left\|{\dot{\theta }}_{e}\right\|}_{\infty }}\right]$$ (17)

subscripts *e* and *s* denote experimental and simulated trials, respectively. Rather than conducting one cost calculation for the entire trial, we further parsed the trials into individual cycles to minimize the effect of frequency variations in the experimental trials on cost *C*. After aligning the peaks of the experimental and simulated cycles (approximately 8 cycles per trials), the absolute difference was calculated between the experimental and simulated trajectories, and then normalized by its H∞ norm of the respective trajectory for that specific trial. *C* was the average cost across all cycles for a given experimental trial for a given combination of *K* and *B*.

This optimization procedure first aimed to identify the combination of *K* and *B* that best approximated each experimental trial. Then, the two-dimensional cost landscapes of all individual 1100 successful trials of all subjects were averaged to minimize the effect of local minima in a cost landscape. This produced one averaged cost landscape as shown in Fig 11B. The minimum cost was found for *K* = 40 N/m and *B* = 40 Ns/m, highlighted by the red point. Hence, these *K* and *B* values were used in all forward simulations.

We also investigated whether the values changed with practice. However, no significant trends were identified.

### Statistical analysis

The trials in which the subjects lost the ball were eliminated from further analysis, as reported in Fig 3. In total, there were 220 failed trials, leaving 1100 successful trials for analysis. In 18 of these failed trials the ball was lost in the preparation interval, in 59 of the failed trials the ball was lost in the first 2 cycles of the rhythmic movement.

The main performance metrics showed approximately exponential time courses over the 120 trials. Therefore, exponential fits were applied to estimate the time constants of these changes. This allowed comparisons of the time scales of these processes to estimate their correlation or dependency. For the changes in initial ball angles, individual fits were computed and the asymptotes were compared. To statistically test whether these changes with practice were significant, the mean values of the first 5 successful trials were compared with the last 5 successful trials of the 11 subjects, using paired *t*-tests within subjects. Note that these 5 trials were the successful ones, excluding failed trials. Hence, the mean especially for the first 5 trials often extended beyond trial number 5, as more than 50% of the subjects had failed trials at the beginning of the experiment (Fig 3A). Prior to the *t*-tests, the data were evaluated for normal distribution using Kolmogorov-Smirnov tests [114]. All dependent measures satisfied the criteria for normal distribution. For the nonlinear changes across trials, the exponential function $y=A(1+e^{-\frac{t}{\tau }})+B$ was used; for increasing time courses, the exponential function $y=A(1-e^{-\frac{t}{\tau }})+B$ was applied. All data processing and analyses were performed with MATLAB (The MathWorks, Natick, MA). The time constants were estimated and the *R*^*2*^ values were computed using the Curve Fitting Toolbox in MATLAB.

### Additional analysis

#### Inverse dynamics: Effect of initial ball velocity on interaction forces

**Fig 12:**
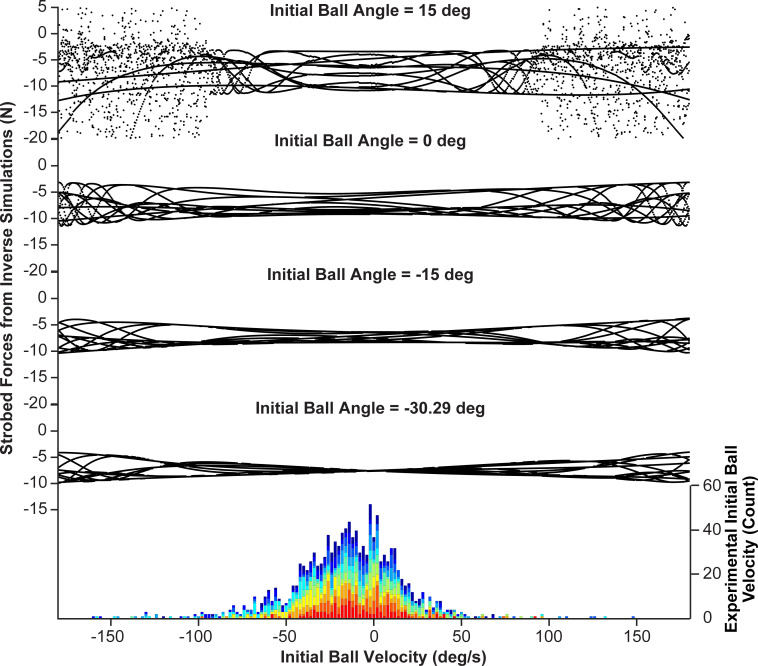
**Effect of initial ball velocity on interaction forces:** Stroboscopic plots of *F*_*inter*_(*t*) across different initial ball velocities, ${\dot{\theta }}_{0}$, shown separately for four different initial ball angles, *θ*_0_; from top to bottom: +15 deg, 0 deg, -15 deg and -30.29 deg. These distributions were obtained for each simulation with different ${\dot{\theta }}_{0}$ from -180 deg/s to +180 deg/s (step size 5 deg/s). The bottom panel includes the histogram of ${\dot{\theta }}_{0}$ measured in all trials pooled over all subjects; different subjects appear in different colors.

To examine the effect of initial ball velocity $\dot{\theta_{0}}$ on *F*_*inter*_(*t*), the same inverse dynamics calculations and stroboscopic summary of force profiles were conducted again, but now for fixed initial ball angles *θ*_0_, while sweeping through a large range of $\dot{\theta_{0}}$ between -180 to +180 deg/s. The initial condition for the cup position *x*_0_ was set to -0.15 m, the left-most position for the cup, and cup velocity was set to ${\dot{x}}_{0}=0$. Four stroboscopic force diagrams were produced for four values of initial ball angles: *θ*_0_ = +15, 0, -15, -30.29 deg. These values were chosen because they represented values of the observed initial ball angles *θ*_0_ and produced a range of different *F*_*inter*_(*t*) behaviors. The value *θ*_0_ = +15 deg was chosen to elicit chaotic behavior. Fig 12 shows the four strobed force distributions for the four different initial ball velocities.

Comparing the structure of the strobed force distributions across the four initial ball angles shows that for *θ*_0_ = +15 deg and 0 deg, there were no values $\dot{\theta_{0}}$ that would produce a simple sinusoidal *F*_*inter*_(*t*). In contrast, the stroboscopic forces for both *θ*_0_ = -15 deg and *θ*_0_ = -30.29 deg were symmetric about $\dot{\theta_{0}}$ = 0 deg/s and had less range in the strobed *F*_*inter*_(*t*) values. The most predictable profile was observed for ball velocity $\dot{\theta_{0}}$ = 0 deg/s at these initial ball angles. For *θ*_0_ = -15 deg and *θ*_0_ = -30.29 deg, the regions with relatively simple interaction forces were -20 < ${\dot{\theta }}_{0}$ < +20 deg/s. Furthermore, for the stroboscopic plots with *θ*_0_ = -15 deg and *θ*_0_ = -30.29 deg, there were no $\dot{\theta_{0}}$ values that would render the profile of *F*_*inter*_(*t*) chaotic. These values best represent the initial ball angles that subjects chose (Fig 8).

#### Comparison with human data

At the bottom of Fig 12, a histogram of the experimental $\dot{\theta_{0}}$ values of all subjects is plotted, each subject in a different color. As summarized above, all subjects converged to ${\dot{\theta }}_{0}$ = -15.81 deg/s on average over the course of their 120 trials, seen in the relatively broad peak of the distribution. If the subjects chose an initial ball angle that was close to -30.29 deg, there were no $\dot{\theta_{0}}$ values that would render their *F*_*inter*_(*t*) profile chaotic. However, the strobe plots with ball angles at -15, 0, and +15 deg did not afford any solution with a simple sinusoidal *F*_*inter*_(*t*).
